# Microbiome analysis reveals the abundance of bacterial pathogens in *Rousettus leschenaultii* guano

**DOI:** 10.1038/srep36948

**Published:** 2016-11-15

**Authors:** Sunil Banskar, Shrikant S. Bhute, Mangesh V. Suryavanshi, Sachin Punekar, Yogesh S. Shouche

**Affiliations:** 1 Microbial Culture Collection, National Centre for Cell Science, Pune, India; 2 Department of Zoology, Savitribai Phule Pune University, Pune, India; 3 Biosphere, Eshwari, 52/403, Lakshminagar, Parvati, Pune-411009 (Maharashtra), India

## Abstract

Bats are crucial for proper functioning of an ecosystem. They provide various important services to ecosystem and environment. While, bats are well-known carrier of pathogenic viruses, their possible role as a potential carrier of pathogenic bacteria is under-explored. Here, using culture-based approach, employing multiple bacteriological media, over thousand bacteria were cultivated and identified from *Rousettus leschenaultii* (a frugivorous bat species), the majority of which were from the family *Enterobacteriaceae* and putative pathogens. Next, pathogenic potential of most frequently cultivated component of microbiome i.e. *Escherichia coli* was assessed to identify its known pathotypes which revealed the presence of virulent factors in many cultivated *E. coli* isolates. Applying in-depth bacterial community analysis using high-throughput 16 S rRNA gene sequencing, a high inter-individual variation was observed among the studied guano samples. Interestingly, a higher diversity of bacterial communities was observed in decaying guano representative. The search against human pathogenic bacteria database at 97% identity, a small proportion of sequences were found associated to well-known human pathogens. The present study thus indicates that this bat species may carry potential bacterial pathogens and advice to study the effect of these pathogens on bats itself and the probable mode of transmission to humans and other animals.

Bats are among the most diverse organisms of the animal kingdom with some very unusual adaptations like flying and echolocation. Bats feed on nectar, fruits, flowers, leaves, insects, small animals and blood, exhibiting their versatile feeding behavior. They offer invaluable services to the ecosystems; they are important pollinator and seed dispersers, thus help in regeneration of deserted lands and also helps in maintaining forest tree diversity^1^,^2^. Further, insectivorous bats also serve as natural pest control agents clearing several tons of insect pests in a single night and saving millions of dollars otherwise required in agricultural pesticides^3^. Consequentially, it helps to reduce the ill-effects of the chemical pest control agents as well.

On the contrary, bats are considered as the reservoir of viruses. The source of many viral outbreaks can be traced back to the bats suggesting that the bats can be a vector of various viral diseases^4^. The recent outbreaks include SARS (Severe Acute Respiratory Syndrome)^5^, MERS (Middle East respiratory syndromes)^6^ and the most recent one Ebola virus outbreak^7^. While their role in virus transmission is well-established^8^, bats have also recognized as a potential source of fungal^9^,^10^ and bacterial pathogens^11^,^12^. Previously, it has been reported that tourists and visitors those who often visited the bat harboring caves or the houses having bats colonies, were contracted with the Histoplasma infection^9^. Further, it has been indicated that bats blood can harbor *Bartonella myotimonensis* which is considered as an important human pathogen. These pathogen carrying bats can transmit these bacteria via their ectoparasites to other animals, therefore, may cause zoonosis^12^.

Although the direct encounter of bats and humans is rare, the bat guano which is excreted beneath and nearby roosting sites of bats could be a potential source of zoonosis. Moreover, bats can fly several kilometers while foraging and may excrete as many as 60 times a day due to their huge diet^10^ and they are reported to defecate even during the flight^13^ hence, may prove unsafe. Previously, many bacterial pathogens have been isolated from apparently healthy bats including from their guano^14^.

Bacterial isolation and identification studies from bats revealed that the bacterial load is in the range from 10^5^ to 10^15^ CFUs^15^,^16^, which suggests they must be harboring a huge number of bacterial species. Therefore, despite of previous reports of bacterial identification from bat guano or the bats intestinal content^17^,^18^ there remains an immense scope of identifying additional bacterial species from bats. Hence, a serious need of detailed and thorough study of bat guano associated bacteria was experienced. Such studies are important to catalogue the various bacteria from bats, so that their potential role in zoonosis can be recognized, interpreted and can be comprehended during the zoonosis.

Metagenomic studies using 16 S rRNA gene sequencing have been used to characterize gut microbial communities of different bats species^19^,^20^. A report from Philips *et al.*^19^ mentioned that, the herbivory dietary strategy and the reproductively active bats carried more diverse microbiota than carnivore and reproductively inactive individuals^19^. Similarly, the Carrillo-Araujo *et al.* reported the diet to be a primary factor to define the gut microbiome of bats^20^. Further, our previous study reported the considerable similarity of microbial communities of frugivorous and insectivorous bats indicating that there was dietary overlap among the bats of different feeding habits^21^. In addition to the diet, the decaying guano communities were also explored revealing a higher abundance of bacterial communities involved in nitrogen cycling^22^. Interestingly, using the same approach, Veikkolainen *et al.*^12^ reported and confirmed the presence of important human bacterial pathogen *Bartonella mayotimonensis*^12^. There are more than 1230 bats species worldwide^21^ which should be explored for their microbial communities to understand a comprehensive ‘bats’ microbiome’ from every aspect including host-microbe relationship as well as their possibility of carrying putative bacterial pathogens.

This study deals with the isolation and identification of general bacterial communities and potential bacterial pathogens present in *Rousettus leschenaultii* guano. Further, to evaluate the pathogenic potential, virulence genes were positively identified from the most commonly isolated bacteria from the gut microbiome i.e. *Escherichia coli.* Additionally, 16 S rRNA gene sequencing was performed using Ion Torrent PGM to identify the bacterial communities and screened for the presence of putative human pathogens. Therefore, this study discourages the human interference in the wildlife which otherwise may cause irreparable loss to the ecosystem and severe human health hazards.

## Results

### Bat guano collection

Fresh bat guano pellets were collected at the Robber’s Cave, Mahabaleshwar, Maharashtra, India. Collection plates were left inside the cave for two hours to capture the guano dropping. This way, a total of 31 fresh guano pellets were collected from which 17 guano of sufficient quantity and free from contaminations were used for the further study.

### Bat Identification

Sequence analysis of the cytochrome B (*cytB*) gene amplified from total extracted DNA of the guano pellets revealed that the collected fecal pellets were from a single bat species i.e*. Rousettus leschenaultii,* a predominant bat species reported from the Robber’s cave^23^. This confirmed that the collected guano pellets were from single bat species.

### Total viable counts and Identification of bacteria

The total viable count of bat guano was performed, it ranged from 1.71 × 10^7^ to 3.34 × 10^10^ CFUs/gram of the guano among four different bacterial media used. All bacterial isolates were identified by 16 S rRNA gene sequencing followed by the BLAST analysis at NCBI. A total of 922 bacterial isolates from bat guano and 143 bacterial isolates from cave environment samples (total 1065) were identified (the entire list of 1065 bacteria identified is provided in Supplementary Table S1). All the isolates showed 99% or above similarity except five isolates.

**Figure 1:**
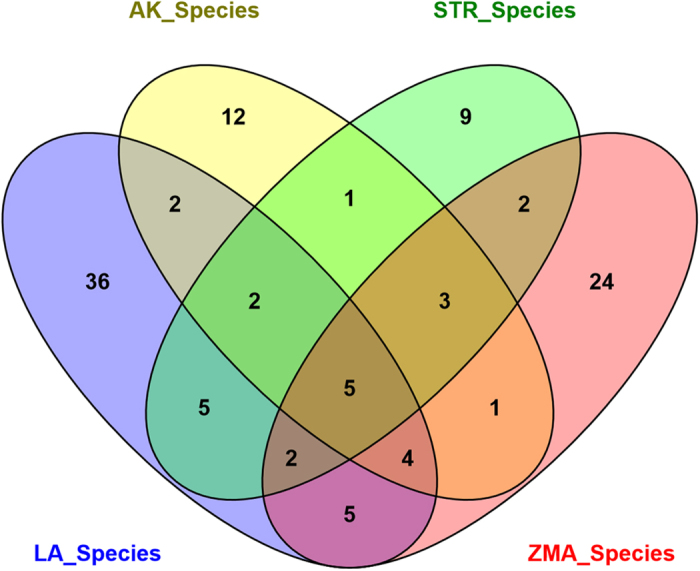
Venn diagram showing the distribution of bacterial species obtained from different bacteriological media. LA; Luria Hiveg Agar, STR Streptomycetes Isolation Agar, ZMA; Zobells Marine Agar, AK; Arret & Kirshbaum medium.

**Figure 2:**
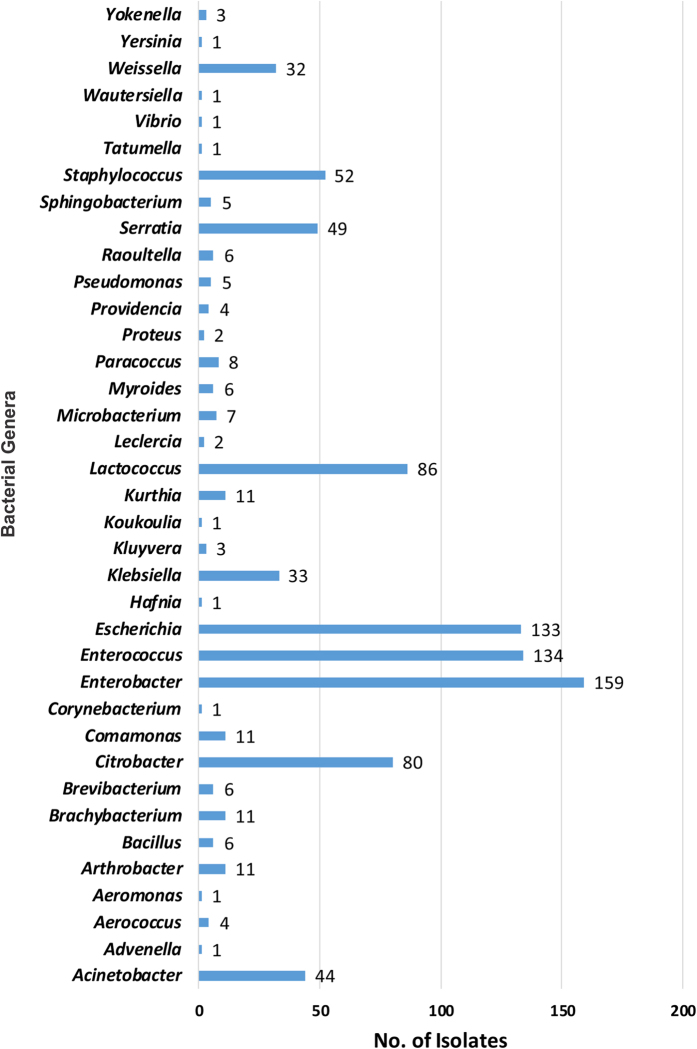
Genus level distribution of bacterial isolates obtained from fresh bat guano.

All 16S rRNA gene sequences of isolates were further analyzed for media-wise genus level distribution. Maximum numbers of bacterial genera (i.e. 26) were obtained using Luria HiVeg Agar (LA) media followed by Zobell’s Marine Agar (ZMA) (21 genera), Arret and Kirshbum agar (AK) (19 genera) then Streptomycetes Isolation Agar (STR) (12 genera). Similarly, LA was found to be the most efficient in capturing unique bacterial genus (7) followed by ZMA (3) then AK (2) and STR (1) (Fig. 1). Thus, total 105 species (Supplementary Figure S2) belonging to 37 different genera (Fig. 2) and 4 bacterial phyla *i.e.* Proteobacteria, Firmicutes, Actinobacteria and Bacteroidetes were isolated from bat guano. The most dominating bacterial genera obtained was *Enterobacter* (151 isolates) followed by *Enterococcus* (134 isolates) and *Escherichia* (133 isolates). Three unique bacterial genera i.e. *Aquitalea*, *Cedecea* and *Pontoea* were obtained from CW (Cave stream Water) sample but not obtained in any other samples whereas only one bacterial genera i.e. *Staphylococcus* was obtained from BCS (Bats’ cave Ceiling Soil) sample (Supplementary Figure S3).

### Literature survey for bacterial pathogens

As taxonomic affiliation of most of the isolates (~60%) was Proteobacteria and majority of pathogens belong to phylum Proteobacteria, we speculated that guano could contain a higher abundance of pathogenic bacteria. Hence, a detailed literature survey was performed for all 105 bacterial species obtained from *R. leschenaultii* guano, to identify their pathogenic potential. Only bacteria belonging to risk group-2 or above were considered a pathogen. This survey revealed that only two bacteria belongs to RG (Risk Group) 2 (Table 1), although other 54 bacterial species were also reported to have association with different opportunistic infections.

### Identification of virulence genes in *Escherichia coli* strains

**Figure 3:**
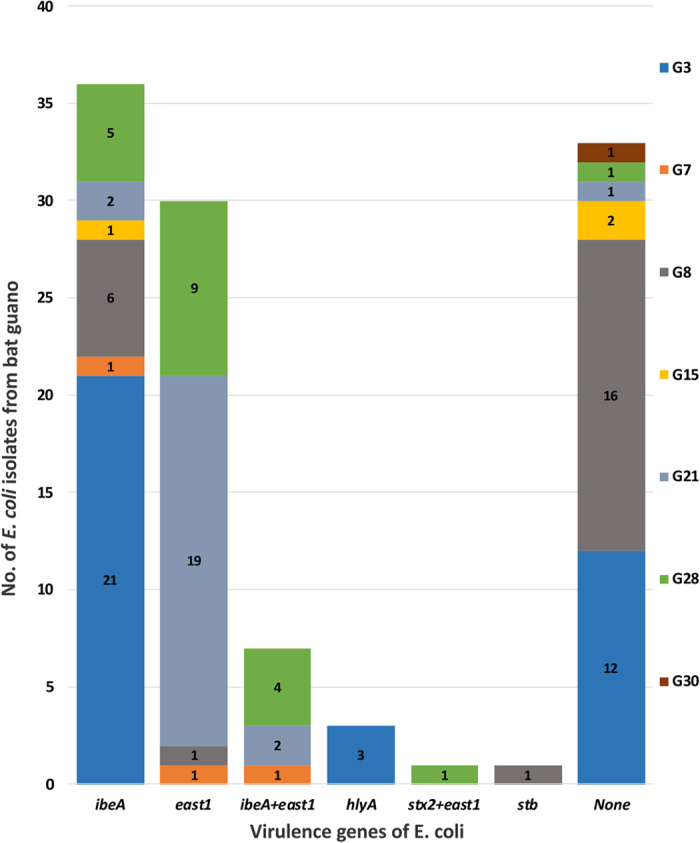
*Escherichia coli* isolates of *R. leschenaultii* guano carrying different virulent genes belonging to different pathotypes. None of the isolates were positive for *ipaH*, *sta* and *stx1* gene. *E. coli* isolates G8ZM15 and G28BLA40 carried *stb* and *stx2* genes. (*hlyA* = gene for alpha hemolysin, *ibeA* = Invasion of brain endothelium (extraintestinal *E. coli* (ExPEC)); *east1* = heat-stable enterotoxin (entero-aggregative *E. coli* (EaggEC)); *stx1* = Shiga toxin-1, *stx2* = Shiga toxin-2 for enterohemorrhagic *E. coli* (EHEC) identification; *ipah* = Invasion Plasmid antigen enteroinvasive *E. coli* (EIEC); *stb* = Heat stable toxin-b, *sta* = Heat stable toxin-a enterotoxigenic *E. coli* (ETEC)).

To cause an infection successfully, bacteria must possess some virulence factors. In this study, *E. coli* was represented by more than 10% of total isolates. Although, it is a common component of a healthy gut microbiome, many virulent strains of *E. coli* (pathotype) also exist. Hence, 96 *E. coli* isolates were assessed for their pathogenic potential by the identification of eight virulence genes (Supplementary Table S4). This assessment revealed 40.6% of isolates to be *ibeA* and *hlyA* gene positive (indicative of extraintestinal pathogenic *E. coli* (ExPEC)) and 31.3% were *east1* gene positive (indicative of enteroaggregative *E. coli* (EAEC)). Additionally, one isolate possessed the enterotoxigenic *E. coli* (ETEC) heat-stable enterotoxin-b (*estII*) gene. Interestingly, one of the *E. coli* isolates was Entero-hemorrhagic (EHEC) bearing Shiga toxin-II (*stx*_*2*_) gene which additionally co-harbored *east1* gene. Seven other cultivated isolates were found positive for *ibeA* and *east1* genes (Fig. 3) while none of the *E. coli* isolates belonged to enteroinvasive (EIEC) pathotype (*ipaH* negative).

On comparison with the previous studies on avian pathogenic strains of *E. coli*^24^, a higher prevalence of the *ibeA* gene was revealed in this study (37.5% vs previous ~26%). Nonetheless, *E. coli* strains isolated from human vagina and neonatal meningitis appears to have similar prevalence of *ibeA* gene^25^,^26^. In present study the prevalence of *east1* gene in *E. coli* strains was quite similar to the previous study (31.3%) in weaned pigs with the diarrheal infections^27^. Thus, a comparable frequency of virulent genes was observed.

### Ion Torrent sequence analysis

**Figure 4:**
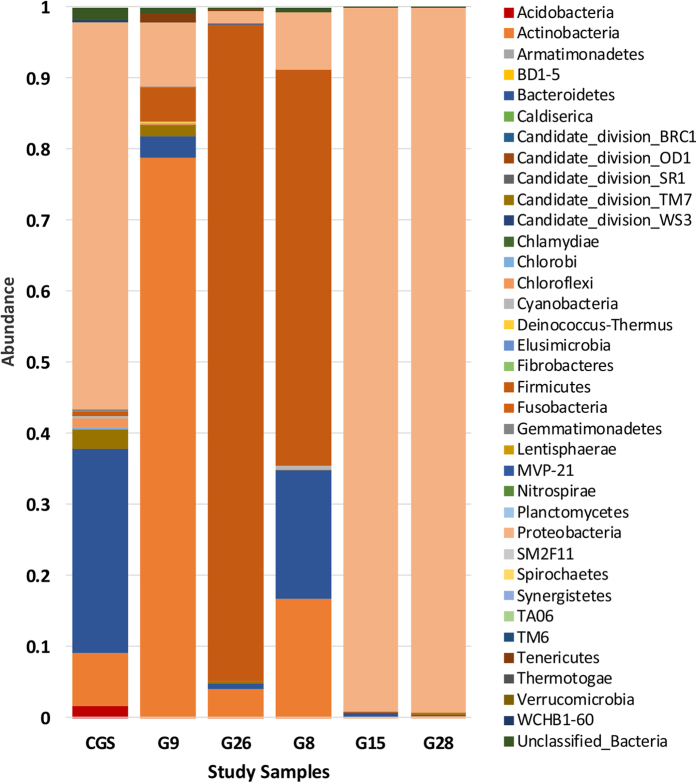
Phylum level distribution of Ion Torrent sequences of different guano samples. CGS = Cave Ground Surface Soil; G = Guano.

**Figure 5:**
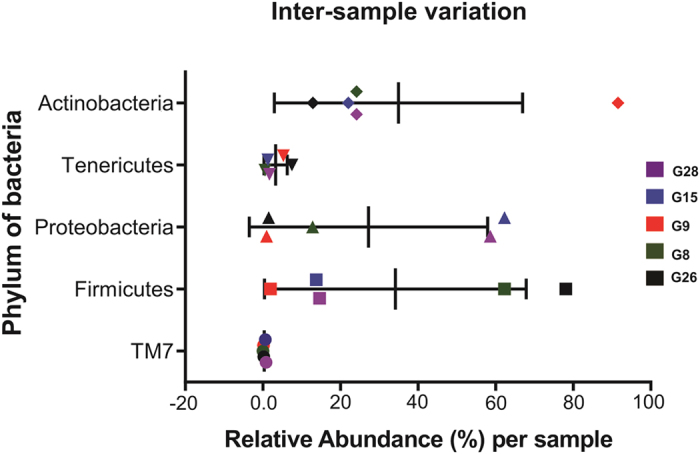
Inter-sample variation in gut microbiome (calculated from bacterial phyla constituting core microbiome). Different color represents the different samples. Different shapes indicate the different phylum of bacteria. The error bars indicate the standard deviation of mean of relative proportion of Ion Torrent sequences.

Further, to get an in-depth overview of total bacterial communities of *R. leschenaultii* guano, 16S rRNA gene sequencing was performed using Ion Torrent PGM. The generated sequences were analyzed using QIIME (Quantitative Insight into Microbial Ecology) package^28^. The results indicated the presence of 31 different bacterial phyla in bat guano which revealed the huge uncultivated bacterial communities present in the bat guano (Fig. 4). Samples G15 and G28 showed the abundance of Proteobacteria (~98%) while G9 had the abundance of Actinobacteria (~78%) and G26 and G8 had the abundance of Firmicutes (92% and 55% respectively). The Cave Ground Surface soil (CGS) sample showed the abundance of Proteobacteria (~54%) followed by Bacteroidetes (~28%) and Actinobacteria (~7.4%). Further, a huge inter-individual variation in bacterial communities was also observed. The various indices of alpha diversity indicated the CGS to be the most diverse sample, whereas, among fresh guano samples G9 had maximum bacterial diversity (Supplementary Figures S5 and S6). Further, computation of core microbiome from Ion Torrent sequences of fresh guano revealed the presence of five bacterial phyla *i.e.* Proteobacteria, Tenericutes, Candidate division TM7, Firmicutes and Actinobacteria but in different proportions (Fig. 5, Supplementary Figure S7).

### Bat Guano Microbiome Comparison

**Figure 6:**
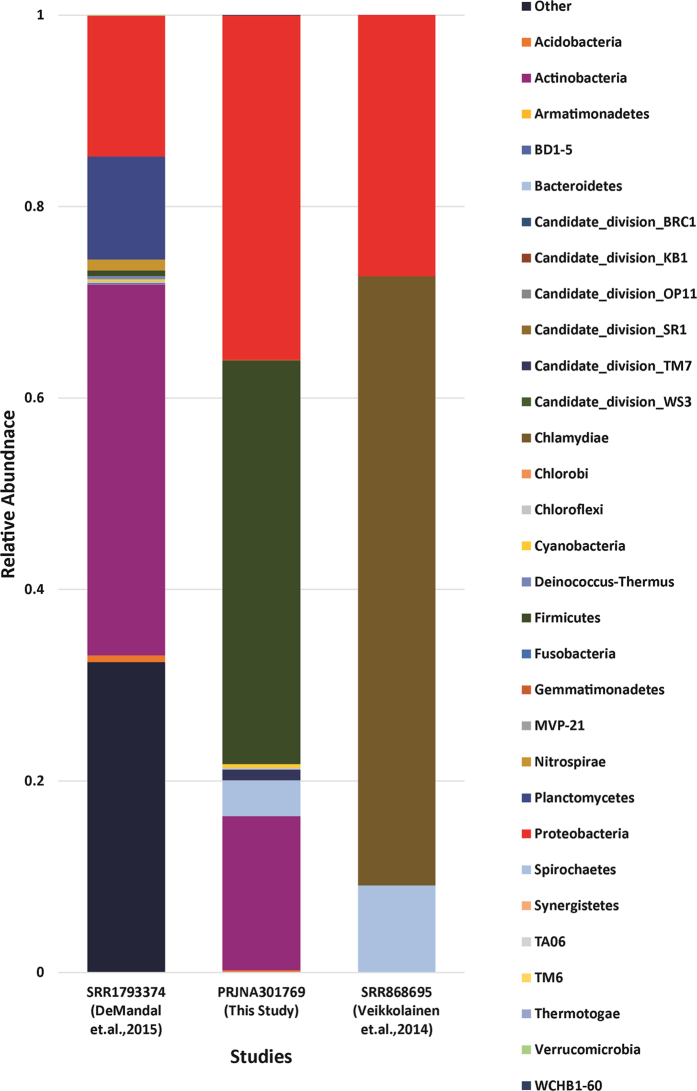
Phylum level distribution of different microbiome studies compared.

Two previous bat microbiome studies were compared with the present bat guano microbiome study. To accomplish this, fastq files were retrieved from SRA (Sequence Read Archive) NCBI, quality filtered and analyzed using QIIME pipeline. The analysis revealed that these three bat microbiome studies are quite different in terms of composition and the relative proportion of bacterial communities. Only three phyla i.e. Chlamydiae (~63.6%), Proteobacteria (~27.3%) and Bacteroidetes (~10%) were represented in Veikkolainen *et al.*^12^ study (location: Finland; bat species: *Myotis daubentonii*). On contrary, the study by De Mandal *et al.*^22^ (location: India; bat species: not available) and present study (location: India; bat species: *R. leschenaultii*) comprised of 21 and 27 bacterial phyla predominated by Actinobacteria (~38.7%) and Firmicutes (~42.1%) respectively (Fig. 6). In an attempt to identify the core bacterial microbiome, at a broader taxonomic level, two bacterial phyla were identified in all three studies (Supplementary Figure S8).

**Figure 7:**
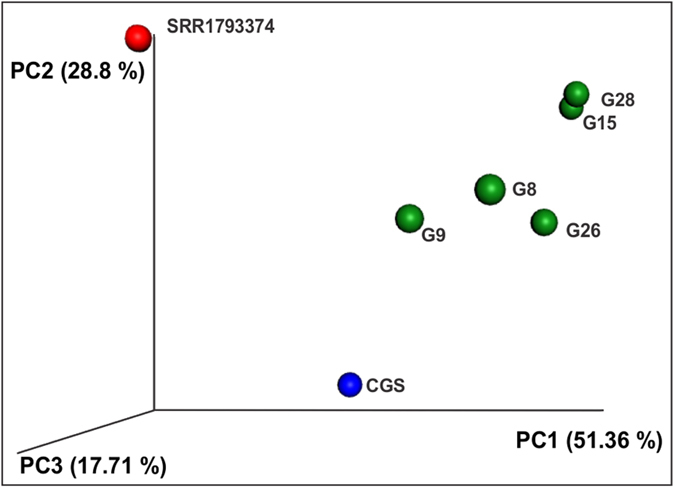
Comparison of composite guano and other samples used in this study. Both representatives of decaying guano (Red and Blue) are far apart from the rest of fresh bat guano samples (green) in PCoA plot generated using the Jaccard distance.

As sample CGS of the present study and composite guano samples used in De Mandal *et al.* study^22^ are representatives of decaying guano hence a comparison between them was performed. The beta diversity analysis using the Jaccard distance revealed that the CGS and the composite guano contained different bacterial communities which in turn were different from the rest of fresh guano samples (Fig. 7).

### Pathogen Identification from Ion Torrent sequences

**Figure 8:**
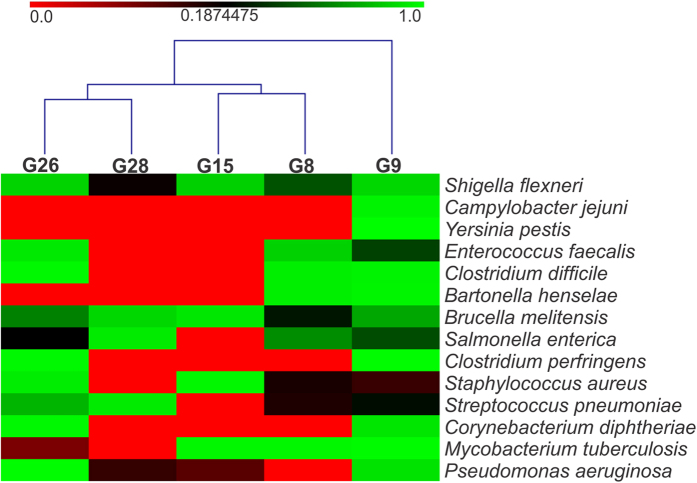
Heat map showing the relative abundance of Ion Torrent sequences identified as bacterial pathogen at 97% sequence identity cutoff.

The presence of potential Bacterial pathogens in culture-based study prompted us to assess Ion Torrent data for their presence. Hence, Ion Torrent sequences were searched against the available human bacteria pathogen database^29^. Upon analysis, at 97% sequence identity cutoff and ≥99% query coverage 2692 (about 0.6504%) sequences showed identity to the well-known pathogens belonging to 14 different species of bacteria including *Yersinia pestis, Brucella melitensis* and *Mycobacterium tuberculosis* etc. (Fig. 8).

### Nucleotide sequence accession number

The Nucleotide sequences of all 1065 bacterial isolates were deposited to NCBI GenBank under the accession numbers [KT260213 - KT260379, KT260380-KT260787, KT260788-KT260998, KT260999-KT261277]. The Ion Torrent sequence reads were also submitted at SRA NCBI under the accession number [SRP066080]. The representative nucleotide sequences of *E. coli* virulence gene were also submitted at European Nucleotide Archive (EMBL) for the accession numbers (LT622255-LT622257, LT622259).

## Discussion

Bats are crucial and the integral part of healthy ecosystem, serving it in many ways such as chiropterophily, a mode of pollination exclusively carried by the bats. Apart from their ecological services, the bio-fertilizers made from the bat guano serve as the rich source of nutrients for better crop production, farming and gardening. Bats also has promising prospects for human health. ‘Draculin’ (Desmoteplase), a glycoprotein from the vampire bat’s saliva, a natural anti-coagulant, is currently in trial to be used as the medication to treat patient of ischaemic stroke. It can reopen the clogged blood vessels so that the damage can be prevented^30^ hence, it can prove to be a life saver.

Despite grandness of bats their every aspect including their gut microbial communities need to be addressed. The present study has attempted to identify the bacterial communities present in *Rousettus leschenaultii’s* (a frugivorous bat species) guano using culture-based approach and has led to the identification of about a thousand bacteria belonging to more than a hundred different bacterial species. A majority of them have been isolated for the first time from bats. Since all cultivated bacteria were identified using nearly full length 16 S rRNA gene sequencing, therefore, identity was ensured. Additionally, Ion Torrent sequencing of 16 S rRNA gene provided information about the general bacterial communities with specific emphasis on pathogens among the total communities present in bat guano.

Our observation of bacterial count for the *R. leschenaultii* guano is in the range of 1.71 × 10^7^–3.34 × 10^10^ CFUs/gram, which is higher than previously reported count i.e. 2 × 10^5^ for the guano of *Myotis* sp. of bats; possibly the use of fresh guano here as against the old and dried pellet used previously could be the reason^31^. The reported bacterial count for the stomach and intestinal contents of different species of bats was in the range of 10^5^–10^15 ^CFUs^10^,^15^,^16^ indicating that the main differences, apart from the sample itself, could be because of inter-individual variation due to host species, diet, body size, specificity and geographical location^32^.

So far, most studies have used one or two bacteriological media to identify the bacterial communities associated with bats^15^,^31^, hence were confined. Few of such studies have reported about 17, 26 and 25 bacterial species from 4, 6 and 10 species of bats respectively^17^,^32^,^33^. In contrast, using four different bacteriological media, we were able to acquire four different bacterial phyla belonging to 37 genera and 105 different species, thus, signifying the importance of traditional approach in microbial communities’ exploration. This fact is further strengthened by the presence of unique 66 bacterial species not reported earlier from bats. Interestingly, only a single genus of bacteria *i.e. Staphylococcus* was found in BCS. Therefore, the possibility of BCS contaminating the guano was excluded and assumed that all bacterial species isolated from bat guano are autochthonous community. Such rock dwelling *Staphylococcus* has been associated with Manganese mobilization in basalt rocks^34^, hence, its high abundance in BCS is explained. While guano shared some bacterial genera with those observed in CW and CGS; the possibilities of these contaminating the guano was taken care during the sampling.

The next generation sequencing platforms are rapidly changing the ways of characterizing microbial community from various sources. Metagenomic studies using these sequencing technologies have contributed enormously to our understanding of structure and functioning of a given system. Accordingly, using Ion Torrent sequencing, it was observed that two guano samples has more than 98% sequences representing the Proteobacteria, one showed the higher abundance of Actinobacteria and another one has the Firmicutes in abundance. Previous studies have demonstrated the huge variation in gut bacterial communities^22^ even in genetically similar human individuals^30^. Therefore, the major reason appears to be the host specificity, individual’s diet, health and physiological state^27^.

Recently, bat guano microbiome was reported from the composite guano collected from cave floor^22^, hence, no information was available about the host bats. Conversely, in addition to multiple guano microbiomes, we identified the bat to be *Rousettus leschenaultii*, a most dominating frugivorous bat in Robber’s Cave^23^. Additionally, alpha diversity and rarefaction analysis indicated the significant microbial enrichment in CGS sample indicating that the decaying process may have led to the increased bacterial diversity^35^. The decaying or the disintegration process is affected by the various physical and environmental factors. Additionally, the biogeochemical processes crucial for the recycling of the organic and inorganic matters are primarily carried out by the microorganism which causes the change in pH (due to the release of ammonia) and nutritional contents which further allow the growth of diverse bacteria^35^. Hence, the enrichment of bacterial communities in the decaying guano is explained. Further, the underlying soil may also cause an increase in the microbial load. The comparison between composite guano and samples from this study revealed that both CGS and composite guano are quite dissimilar in microbial composition from each other as well as rest of the fresh guano samples which further indicates the bacterial communities of decaying guano is rather different from the fresh guano. We speculate that the differences in two different decaying guano could be because of their origin, host bats’ species, duration of the decaying process and other physicochemical properties leading to the different trajectories of development of microbial communities.

At the broader taxonomic assignments, we were able to detect 31 bacterial phyla as against only 18 phyla in composite guano samples^22^. While this holds true, the core microbiome contained only five different phyla: Actinobacteria, Candidate division TM7, Firmicutes, Proteobacteria and Tenericutes, which indicates probably all fresh guano of *R. leschenaultii* are characterized by the presence of these five phyla. We believe that the other bacterial phyla that we were able to recover could be derived from the consumed food material, as bats have a huge diet which remains only transiently in the gastrointestinal tract^10^. Further, on a comparison between Ion Torrent sequencing results and culture based finding, it was observed that three major bacterial phyla i.e. Actinobacteria, Firmicutes and Proteobacteria were shared confirming the stable nature of these bacterial phyla in bat gut environment. A higher abundance of Actinobacteria was observed in Ion Torrent sequence data, whereas, the higher relative proportion of Firmicutes and Proteobacteria was obtained in culture, suggesting fewer Actinobacteria were cultivable on the employed media. Similarly, due to the stringent growth requirements, Tenericutes could not be obtained in culture^36^ even though it was one of the members of core communities. Furthermore, an absence of Bacteroidetes in the core microbiome and its presence in culture indicated the individual variations of guano samples.

Comparison of the present study with the two previously published microbiome studies revealed that this study reported a higher number of bacterial phyla, probably the fresh guano samples collected for the study appears to be the prime reason. Further, in an attempt to find the core microbiome from different microbiome studies indicated that only two phyla i.e. Proteobacteria and Bacteroidetes are present commonly in three compared studies. The other two recent microbiome studies showed the strong influence of host phylogeny, dietary pattern, physiology and geography on the gut microbial communities of bats^19^,^20^.

Earlier bats have been suspected as the reservoir of the human bacterial pathogens^12^ and their guano was considered important in pathogen dissemination^37^. Here, two bacterial species i.e. *Escherichia coli* (Risk Group-I/II) and *Staphylococcus aureus* (Risk Group-II) were cultivated. Additionally, 54 bacterial species obtained from guano have been reported to cause various human infections. Except *Acinetobacter johnsonii* (cause fish infection) and *Staphylococcus lentus* (cause mastitis in goats) others were mainly found involved in the sepsis, urinary tract infections (UTIs) and other infections especially in immune compromised individuals.

In the present study, a majority of the cultivated bacteria belong to the family *Enterobacteriaceae* and most of these isolates were identified as *Escherichia coli* (116, ~12.6%), a well-known commensal gut inhabitant and opportunistic RG-I/II pathogen^38^. Hence, a high percent of ExPEC and EaggEC pathotype in *R. leschenaultii* guano could be a serious health concern. Additionally, shiga toxin producing strain has been linked with the severe outbreak in the Germany, leading to more than 4000 cases and more than 50 deaths^39^. These results are quite staggering, indicating that the *E. coli* recovered from *R. leschenaultii* guano may prove to be pathogenic.

Few studies have indicated the presence of virulence genes in wild animals^40^,^41^. Cabal *et al.*^42^ compared the virulence gene profile of *E. coli* isolated from cattle, swine and broiler. The results indicated the presence of *stx1* and *stx2* gene (from both O157:H7 and Non-O157:H7 *E. coli* serotypes) in cattle and swine^42^. Similarly, post weaning diarrhea in piglets is a common problem in piggery farms and the major reason appears to the EPEC/ETEC carrying different virulence genes especially the heat labile/stable toxins (*sta* and *stb*)^43^,^44^.

Recently, even the healthy dairy cows have been reported to carry virulent genes (*stx2, st* and *lt*) in *E. coli* isolated from their dung^45^. Additionally, the humans are reported to be the prime reservoirs for EAEC, EPEC and EIEC^46^,^47^, though they remain healthy. Hence, commensals like *E. coli* may carry virulence genes but often do not cause the infections as the appropriate combination of virulent genes, required to cause the infection, is not available^38^. Likewise, merely the presence of RG-2 bacteria from the host does not indicate it to be the pathogen as all strains of a species may not be the pathogen. Nevertheless, a possibility of gradually acquiring the additional virulence genes, required to cause a successful infection cannot be ignored.

In the light of presence of various pathotypes of *E. coli*, it is essential to discuss other potentially pathogenic bacteria as well. In the discussion that follows numbers in the parentheses (**in bold**) indicate an abundance of the said bacterial species. The other isolated member of genus *Escherichia* is *Escherichia fergusonii* (**16**), a close relative of *E. coli* which has been isolated from the feces of many warm blooded animals. It has also been recovered from the wound infections, UTIs, diarrhea etc.^48^ and reported to cause the bacteremia in diabetic patient^49^. The other dominating genera cultivated was *Enterobacter* e.g. includes *Enterobacter asburiae* (**35**), *Enterobacter ludwigii* (**21**), *Enterobacter cloacae* (**102**) and members of *enterococci* i.e*. Enterococcus faecalis* (**37**) and *Enterococcus hirae* (**81**) which causes various infections especially in immunocompromised individuals^50^,^51^,^52^,^53^. Notably, *E. faecalis* is among the most common species isolated from human clinical samples.

Both *Citrobacter koseri* (**41**) and *Citrobacter freundii* (**29**) are the opportunistic pathogens causing neonatal infections but the neonatal meningitis caused due to *C. koseri* have a high mortality rate^54^. Similarly, *Serratia marcescens* (**24**) is a common cause of nosocomial, urinary tract and wound infections^55^,^56^.

Relatively fewer Gram-positive bacteria were obtained from guano. The main genus among these is *Staphylococcus*. It included *Staphylococcus aureus* (a RG-2 organism), *Staphylococcus nepalensis* (**13**) and *Staphylococcus lentus* (**49**), which are well-known to cause various infections in humans as well as in other animals^57^,^58^. Therefore, it may pose a threat to the herds of sheep and goats grazing nearby bats’ roosting sites.

Similarly, potential bacterial pathogens were also searched in Ion Torrent data using the Human Bacteria Pathogen Database. This database was created from the ‘human pathogenic bacteria virulence factor database’ and only the bacterial species whose proteins were experimentally validated were considered for its creation^29^. Although, the Ion Torrent generates the sequences of shorter read length (here ~200 bp), the use of V3 region for the identification ensured maximum coverage of diversity (~96–99%)^59^. In addition, the only sequence which had high query coverage (≥99%) and high similarity value (>97%) during the BLAST search against the database was considered for the analysis^60^. Hence, the possibility of the presence of these pathogens may not be ignored.

Pathogens identified from Ion Torrent sequencing includes *Pseudomonas aeruginosa*, and *Clostridium perfringens* etc. *P. aeruginosa* (**240**) is a Gram-negative pathogen responsible for several nosocomial infections and opportunistic infections^61^,^62^. The bacterial species *Salmonella enterica* (**347**) and *Shigella flexneri* (**174**) causes gastroenteritis (food poisoning) and shigellosis^63^, respectively. These can be transmitted by the direct contact, fecal or oral route. Hence, the consumption of food contaminated with bat guano should be avoided. The other species found i.e. *Streptococcus pneumoniae* (**375**), *Enterococcus faecalis* (**240**), *Staphylococcus aureus* (**647**) which may cause streptococcal pneumonia, meningitis, bacteremia, infectious lesions, neonatal infections and septicemia^64^,^65^,^66^.

The important observation was the presence of sequences affiliated to *Mycobacterium tuberculosis* (**38**) and *Corynebacterium diphtheriae* (**32**). *Mycobacterium tuberculosis* is an airborne disease and spread through the droplet nuclei. Similarly, *Corynebacterium diphtheriae*, a causative agent of diphtheria, may cause skin infections and septicemia by the droplets, secretions or direct contact^67^. Hence the proper caution should be taken when entering a cave having bats’ roosting site.

Additionally, *Bartonella henselae* (**12**), one of the causative agents of bartonellosis (Cat scratch disease), has been suspected earlier from bats^11^ which can be transmitted to humans by accidental bite or scratches. Additionally, it can also be transmitted by the ectoparasites harbored by the infected animals^12^. Some sequences were also found affiliated to *Brucella melitensis* (**202**) which is the most pathogenic *Brucella* species to humans^68^. It principally affects the goats and sheep, hence, the grazing goats and sheep which comes in contact with bats’ guano may get infected. Hence, these may cause zoonotic infections in humans by direct contact, aerosol inhalation^69^ or indirectly by consumption of improperly cooked food products from diseased animals.

In conclusion, the present study revealed that each gram of *R. leschenaultii’s* guano contains millions of bacteria, belonging to hundreds of different bacterial species, some of which can be potentially pathogenic to humans. Additionally, computed core microbiome revealed five bacterial phyla, three of them are found in the culture-based study. Further, a fairly large inter-individual variation in microbiome composition of guano was observed indicating the individual specificity. The virulence gene profiling of cultivated *E. coli* strains from guano revealed the presence of many virulent genes, though it may not be declared pathogens. This study reports the largest culture-based study from *R. leschenaultii* bat guano to identify the cultivable bacterial diversity and potential human pathogenic bacteria. Thus it is strongly advised that the humans should not interfere with the wild life otherwise it may cause huge loss to the environment and health related issues to the humans.

## Material and Methods

### Sampling site and sample collection

Guano samples were collected from the ‘Robbers cave’ (17°52′57“N and 73°40′35“E) situated in the basalt rock structure of Western Ghats, of Maharashtra state, India. Collection plates were placed on cave ground surface under the roosting bats’ colony and were left for about 2 hours. After 2 hours individual guano pellets were observed and collected separately. Thus, a total of 31 guano pallets were collected and kept at 4 °C for transportation to the laboratory for further processing. Only 17 guano samples with sufficient quantity were used for the bacterial isolation. Other potentially interfering and guano contaminating factors were also included in the study e.g. Cave Water (CW, from a stream which flows from a side of the cave, Cave ceiling soil (BCS, which continuously fall on the ground and hence in guano pellets) and Cave Ground Surface Soil (CGS).

### Bat species identification

Bats were identified using mitochondrial *cytB* gene amplification and sequencing using primers L14724 (5′-CGAAGCTTGATATGAAAAACCATCGTT-3′) and H15149 (5′-AAACTGCAGCCCCTCAGAATGATATTTGTCCTCA-3′), reported previously for the identification of bat species^70^.

### Bacteriological Media Used

Four different bacterial culture media were utilized to capture the maximum bacterial diversity. It includes ZMA (Zobell’s Marine Agar), AK2 (AK Agar No. 2/Arret & Kirshbaum medium), Streptomycetes Isolation Agar (STR), Luria HiVeg Agar (LA) (Himedia Laboratories, India).

### Sample Processing

About the half quantity of all selected samples was suspended in 1 ml of water and mixed thoroughly. The supernatant was serially diluted up to 10^−5^ followed by plating of dilution 10^−4^ in different media plates (dilutions 10^−4^ and 10^−5^ were used for LA media) and incubation for 16–18 hours at 37 °C incubators. Post incubation plates were observed and total colony forming units (CFUs) were counted. Then total viable counts were calculated by considering the number of CFUs observed, dilution factor, the volume of supernatant (inoculum) and the weight of guano used. Subsequently, about 21 random colonies of guano sample from each media were sub-cultured in their respective medium. Post-incubation a loop-full of each colony was suspended in TE (Tris-Ethylenediamine Tetra Acetate) buffer (pH 8.0) to use further for DNA isolation. The remaining guano sample was used for the total bacterial community DNA extraction for Ion Torrent sequencing.

### Bacterial DNA isolation and species identification

For Bacterial identification, DNA of pure cultures was extracted using PureLink ^®^ Pro 96 Genomic DNA Purification Kit (Invitrogen, Inc. USA). PCR amplification of 16 S rRNA gene was performed in triplicates using 10X PCR buffer, 0.2 mmol/L of dNTPs, 0.5 units of *Taq* polymerase (Bangalore Genei) and 10 picomols of each primers 8 F (5′-AGA GTTTGATCCTGGCTCAG -3′) and 1492 R (5′-CGGTTACCTTGTTACGACTT-3′). PCR parameters include initial denaturation at 95 °C for 5 min followed by 1 min at 95 °C denaturation, 55 °C annealing and extension at 72 °C for 35 cycles followed by final extension at 72 °C for 10 minutes then incubation at 4 °C. PCR amplified products were gel checked for positive products. The positive products were purified using 20% PEG-NaCl (Polyethylene Glycol-NaCl). The purified products were processed and sequenced using ABI 3730XL DNA Analyzer (Applied Biosystems, USA). To obtain nearly full-length sequence (1.5 kb) of bacterial 16 S rRNA gene, internal primers, 704 F (5′-GTAGCGGTGAAATGCGTAGA-3′) and 907 R (5′-CCGTCAATTCMTTTGAGTTT-3′) were also used. The sequences were concatenated using ChromasPro V1.4 (http://www.technelysium.com.au/ChromasPro.html), followed by BLAST analysis at NCBI. A BLAST hit, with sequence similarity ≥97% and Query coverage ≥99 is considered as the species of the isolate.

### Identification of Bacterial Pathogens

Only bacterial species belonging to risk group-2 or above were considered pathogen in present study (NIH guidelines; http://osp.od.nih.gov/sites/default/files/NIH_Guidelines.html#_To c446948381).

### Identification of virulence genes in *Escherichia coli*

Eight virulence factor genes (Supplementary Table S4) were used to identify the five different pathotype of the *E. coli*^33^. The DNA extracted from the 96 *E. coli* isolates was used to amplify and check the pathotype of *E. coli.* The PCR was performed using 10X PCR buffer, 0.2 mmol/L of dNTPs, 0.5 units of *Taq* polymerase (Thermo Scientific Inc. USA) and 10–15 picomols of respective primers. PCR parameters include initial denaturation at 95 °C for 5 min followed by 1 min at 95 °C denaturation, different annealing temperatures^33^ for 45 Seconds and extension at 72 °C for 1 minutes for 35 cycles followed by a final extension at 72 °C for 10 minutes then incubation at 4 °C.

### Bat guano community DNA extraction and PCR amplification of 16S rRNA gene

Total community DNA extracted from bat guano using stool DNA isolation kit (Qiagen, the Netherlands), was used for the Ion Torrent analysis. The extracted DNA was quantified using Nanodrop (Nanodrop, Thermo Scientific, USA), followed by PCR amplification of V3 region of 16 S rRNA using hi-fidelity AmpliTaq gold (Invitrogen Inc., USA) and eubacterial universal primers 341 F (5′-CCTACGGGAGGCAGCAG-3′) and 518 R (5′-ATTACCGCGGCTGCTGG-3′). Resulting PCR products were purified using Agencourt AMPure XP DNA purification beads (Beckman Coulter, USA) which were then subjected to end repair. The blunt ended products were used as a substrate for sample-specific barcode and adapter ligation reaction as per the manufacturer’s instructions. Prior to the sequencing, all amplicons were assessed for size distribution and molar concentrations using Bioanalyzer 2100 (Agilent Technologies, USA). The concentration of all the amplicons was adjusted to lowest DNA concentration and subsequently, amplicons were pooled in an equimolar ratio and diluted so as to obtain the pooled amplicons of around 26 pm. The pooled amplicons were attached to Ion Sphere particles (ISPs) and used for emulsion PCR using IonXpress Template-200 kit using IonOneTouch system. Next, template-positive ISPs were enriched using IonOneTouch ES system. The enriched ISPs were then loaded onto 316 chip and sequencing was performed on Ion Torrent Personal Genome Machine (Life Technologies, USA) for 130 cycles.

### Ion Torrent Data analysis

Barcode specific fastq files were processed using mothur pipeline^57^ to obtain fasta and quality file. These two types of the files were quality filtered using following conditions: Size 150–200 bp, homo-polymer max. 5, ambiguity max 0, and average quality score 20. This way we were able to obtain 874999 good quality sequences. All these reads were pooled into a single fasta file and analyzed using QIIME (Quantitative Insight into Microbial Ecology) package. Briefly, OTUs picking was done using open reference approach at 97% sequence similarity cutoff. A representative sequence of each OTU was picked up and lowest possible taxonomic rank was assigned to each of them by using RDP classifier (v2.7)^71^ and SILVA database (SiLVA_111)^72^ as reference. In order to assess the relationship between sequencing depth and discovery of new OTUs, rarefaction analysis was performed. Further, diversity measurements were made using alpha diversity indices such as Shannon, Simpson and Chao1. For computing core OTUs, CGS sample was excluded and a core OTU was defined as an OTU present in all (100% of) the samples.

### Comparison of Different Microbiome Studies

Microbiome data was retrieved from SRA (Sequence Read Archives) NCBI in fastq format to compare present bat guano microbiome studies with the previously published microbiome studies. Thus including ours, a total three studies i.e. De Mandal *et al.*^22^, Veikkolainen *et al.*^12^ were compared. All the files were quality filtered using mothur pipeline and only good quality sequence having a length more than 100 bases and homopolymers allowed no more than 5 bases and having 0 (zero) ambiguity were used in the comparison. Later all the filtered, good quality sequences were compiled into a single fasta file and processed using QIIME pipeline. A closed reference approach using SiLVA_111_database^72^ as reference was used to pick OTUs so that different studies which has utilized the different region of 16 S rRNA gene can be compared.

### Pathogen Identification from Ion Torrent sequencing data

Bacterial pathogen database^29^ was used for the search of the bacterial pathogens from Ion Torrent sequencing reads. All the 16 S rRNA gene sequences of bat guano (excluding CGS) were compiled into a single fasta file (containing 441560 sequences) and searched against the above mentioned database for the sequence similarity using ncbi-blast-2.2.30+ algorithm^73^. Thus, every sequence was assigned the closest hit. Only BLAST hit showing query coverage ≥99% and maximum identity with maximum score value and lowest e-value was considered. The number of sequences having blast hit with ≥97% identity were short-listed and calculated the proportion of sequences affiliated to the bacterial pathogens.

### Statistical analysis

Statistical analysis was performed in Graphpad Prism (v6.0) and the Venn diagram was prepared in the Venny 2.0 (http://bioinfogp.cnb.csic.es/tools/venny/). The heat maps were constructed using MEV software.

## Additional Information

**How to cite this article**: Banskar, S. *et al.* Microbiome analysis reveals the abundance of bacterial pathogens in *Rousettus leschenaultii* guano. *Sci. Rep.* **6**, 36948; doi: 10.1038/srep36948 (2016).

**Publisher’s note**: Springer Nature remains neutral with regard to jurisdictional claims in published maps and institutional affiliations.

## Supplementary Material

Supplementary Table S1

Supplementary Information

## Figures and Tables

**Table 1:** Bacterial pathogens cultivated from *Rousettus leschenaultii* guano having high risk potential.

Sr. No.	Species	Risk Group*	Potential/Known Infection	Reference
1	*Escherichia coli*	I/II	UTI, Endocarditis, diarrhea	(Branger *et al*.^74^; Johnson^75^)
2	*Staphylococcus aureus*	II	Sepsis, Abscess, peritonitis, Health Care associated pneumonia, nosocomial infections	(Lowy^76^; Piraino *et al*.^64^)

*Risk Group as per NIH guidelines. UTI = Urinary Tract Infections; RG = Risk Group.
