# Supplementary material for: Microbiome analysis reveals the abundance of bacterial pathogens in Rousettus leschenaultii guano
Source: Sci Rep. 2016 Nov 15;6:36948. doi: 10.1038/srep36948 (PMC5109407; doi:10.1038/srep36948)
Supplement: Supplementary Table S1 [file srep36948-s1.pdf]

Title: Microbiome analysis reveals the abundance of bacterial pathogens in *Rousettus leschenaultii* guano

Sunil Banskar[1], Shrikant S. Bhute[2], Mangesh V. Suryavanshi[1],Sachin Punekar[3], Yogesh S. Shouche [1*]

Author Affiliations:

[1] Microbial Culture Collection, National Centre for Cell Science, Pune, India.

[2] Department of Zoology, Savitribai Phule Pune University, Pune, India.

[3] Biosphere, Eshwari, 52/403, Lakshminagar, Parvati, Pune-411009 (Maharashtra), India.

* Correspondence:

Dr. Yogesh S. Shouche

Microbial Culture Collection

National Centre for Cell Science,

Central Tower, Sai Trinity Building Garware Circle,

Sutarwadi, Pashan Pune, Maharashtra 411021, India

Fax No: +91-20-25329001

Telephone No: +91-20-25329000

Email: yogesh@nccs.res.in

Sr. No.	Sample Id	Identification	Query cover	Identity
1	G3_AK4	*Escherichia coli*	100%	99%
2	G3_AK5	*Escherichia coli*	100%	100%
3	G3_AK6	*Escherichia coli*	100%	99%
4	G3_AK7	*Escherichia coli*	100%	100%
5	G3_AK10	*Escherichia coli*	100%	99%
6	G3_AK11	*Escherichia coli*	100%	99%
7	G3_AK12	*Escherichia coli*	100%	100%
8	G3_AK13	*Escherichia coli*	100%	99%
9	G3_AK14	*Escherichia coli*	99%	99%
10	G3_AK15	*Escherichia coli*	100%	99%
11	G3_AK16	*Escherichia coli*	99%	99%
12	G3_AK18	*Escherichia coli*	100%	99%
13	G3_AK19	*Escherichia coli*	99%	99%
14	G3_AK21	*Escherichia coli*	100%	100%
15	G7_AK3	*Staphylococcus cohnii*	100%	99%
16	G7_AK4	*Comamonas testosteroni*	100%	99%
17	G7_AK8	*Enterococcus hirae*	100%	99%
18	G7_AK9	*Comamonas testosteroni*	100%	99%
19	G7_AK10	*Lactococcus garvieae*	100%	99%
20	G7_AK15	*Enterobacter cloacae*	100%	99%
21	G7_AK17	*Citrobacter murliniae*	100%	99%
22	G7_AK19	*Staphylococcus cohnii*	100%	99%
23	G7_AK20	*Acinetobacter johnsonii*	100%	98%
24	G7_AK21	*Hafnia alvei*	100%	100%
25	G8_AK2	*Lactococcus garvieae*	100%	100%
26	G8_AK3	*Lactococcus garvieae*	100%	100%
27	G8_AK4	*Escherichia coli*	100%	99%
28	G8_AK5	*Lactococcus garvieae*	100%	99%
29	G8_AK6	*Lactococcus garvieae*	99%	99%
30	G8_AK7	*Lactococcus garvieae*	99%	100%
31	G8_AK9	*Lactococcus garvieae*	100%	99%
32	G8_AK11	*Citrobacter koseri*	99%	99%
33	G8_AK12	*Escherichia coli*	100%	99%
34	G8_AK13	*Escherichia coli*	100%	99%
35	G8_AK14	*Citrobacter freundii*	100%	99%
36	G8_AK15	*Lactococcus garvieae*	100%	99%
37	G8_AK16	*Lactococcus garvieae*	100%	99%
38	G8_AK19	*Lactococcus garvieae*	100%	100%
39	G8_AK20	*Citrobacter koseri*	100%	99%
40	G9_AK1	*Citrobacter koseri*	100%	99%
41	G9_AK2	*Enterococcus hirae*	100%	99%
42	G9_AK3	*Klebsiella oxytoca*	100%	99%
43	G9_AK4	*Klebsiella oxytoca*	100%	99%
44	G9_AK6	*Enterococcus hirae*	99%	99%
45	G9_AK7	*Citrobacter koseri*	100%	99%
46	G9_AK14	*Enterococcus hirae*	100%	99%

47	G9_AK16	*Enterococcus hirae*	100%	99%
48	G9_AK17	*Citrobacter koseri*	100%	99%
49	G15_AK2	*Myroides odoratimimus*	100%	100%
50	G15_AK4	*Proteus hauseri*	100%	99%
51	G15_AK6	*Providencia alcalifaciens*	100%	99%
52	G15_AK7	*Sphingobacterium sp.*	100%	99%
53	G15_AK9	*Aeromonas hydrophila*	99%	99%
54	G15_AK10	*Comamonas testosteroni*	99%	99%
55	G15_AK13	*Staphylococcus kloosii*	99%	99%
56	G15_AK15	*Comamonas testosteroni*	99%	99%
57	G15_AK20	*Klebsiella oxytoca*	100%	99%
58	G17A_1	*Lactococcus garvieae*	100%	100%
59	G17A_2	*Lactococcus garvieae*	100%	99%
60	G17A_3	*Lactococcus garvieae*	100%	99%
61	G17A_5	*Lactococcus garvieae*	100%	99%
62	G17A_7	*Serratia marcescens*	100%	99%
63	G17A_8	*Serratia marcescens*	100%	99%
64	G17A_9	*Serratia ureilytica*	100%	99%
65	G17A_10	*Serratia marcescens*	99%	99%
66	G17A_11	*Serratia marcescens*	100%	99%
67	G17A_12	*Serratia marcescens*	100%	99%
68	G17A_14	*Lactococcus garvieae*	100%	99%
69	G17A_15	*Lactococcus garvieae*	100%	100%
70	G17A_19	*Klebsiella oxytoca*	100%	99%
71	G17A_20	*Lactococcus garvieae*	100%	99%
72	G19A_3	*Kluyvera ascorbata*	100%	99%
73	G19A_5	*Enterobacter cloacae*	100%	99%
74	G19A_7	*Acinetobacter gerneri*	100%	100%
75	G19A_15	*Citrobacter freundii*	100%	99%
76	G19A_17	*Comamonas jiangduensis*	100%	98%
77	G19A_18	*Enterobacter cloacae*	99%	99%
78	G19A_19	*Kluyvera intermedia*	100%	99%
79	G19A_20	*Citrobacter freundii*	99%	99%
80	G25A_6	*Staphylococcus lentus*	100%	99%
81	G25A_7	*Staphylococcus lentus*	100%	99%
82	G25A_10	*Lactococcus garvieae*	100%	99%
83	G25A_11	*Citrobacter amalonaticus*	99%	99%
84	G26A_1	*Lactococcus garvieae*	100%	99%
85	G26A_2	*Lactococcus garvieae*	99%	99%
86	G26A_3	*Enterobacter cloacae*	99%	100%
87	G26A_5	*Lactococcus garvieae*	100%	99%
88	G26A_6	*Lactococcus garvieae*	100%	99%
89	G26A_8	*Lactococcus garvieae*	100%	100%
90	G26A_9	*Lactococcus garvieae*	98%	100%
91	G26A_10	*Lactococcus garvieae*	100%	100%
92	G26A_11	*Lactococcus garvieae*	99%	99%
93	G26A_13	*Lactococcus garvieae*	99%	99%

94	G26A_14	*Enterobacter cloacae*	100%	99%
95	G26A_15	*Lactococcus garvieae*	99%	99%
96	G26A_16	*Lactococcus garvieae*	99%	99%
97	G26A_17	*Lactococcus garvieae*	99%	99%
98	G26A_18	*Enterobacter cloacae*	100%	100%
99	G26A_20	*Lactococcus garvieae*	100%	100%
100	G26A_21	*Lactococcus garvieae*	100%	99%
101	G28A_1	*Raoultella ornithinolytica*	99%	99%
102	G28A_2	*Lactococcus garvieae*	100%	100%
103	G28A_3	*Enterococcus faecalis*	100%	99%
104	G28A_4	*Enterococcus hirae*	100%	99%
105	G28A_5	*Enterococcus hirae*	100%	99%
106	G28A_6	*Enterococcus hirae*	100%	99%
107	G28A_7	*Enterococcus hirae*	100%	99%
108	G28A_11	*Raoultella ornithinolytica*	100%	99%
109	G28A_13	*Enterococcus faecalis*	100%	99%
110	G28A_14	*Lactococcus garvieae*	99%	99%
111	G28A_18	*Enterococcus hirae*	99%	99%
112	G28A_19	*Enterococcus hirae*	100%	99%
113	G28A_20	*Enterococcus hirae*	99%	99%
114	G28A_21	*Raoultella ornithinolytica*	100%	99%
115	G30A_2	*Lactococcus garvieae*	99%	99%
116	G30A_4	*Lactococcus garvieae*	100%	99%
117	G30A_5	*Lactococcus garvieae*	100%	100%
118	G30A_6	*Serratia marcescens*	100%	99%
119	G30A_7	*Lactococcus garvieae*	100%	99%
120	G30A_8	*Enterococcus faecalis*	100%	99%
121	G30A_9	*Raoultella planticola*	100%	99%
122	G30A_13	*Lactococcus garvieae*	100%	99%
123	G31A_1	*Enterobacter hormaechei*	100%	99%
124	G31A_2	*Lactococcus garvieae*	99%	100%
125	G31A_3	*Lactococcus garvieae*	100%	100%
126	G31A_4	*Lactococcus garvieae*	100%	100%
127	G31A_9	*Citrobacter freundii*	99%	99%
128	G31A_13	*Lactococcus garvieae*	100%	100%
129	G31A_14	*Citrobacter freundii*	100%	99%
130	G31A_16	*Lactococcus garvieae*	99%	100%
131	G31A_18	*Lactococcus garvieae*	99%	100%
132	G31A_19	*Lactococcus garvieae*	100%	100%
133	G31A_21	*Brachybacterium tyrofermentans*	99%	99%
134	BCSA_1	*Staphylococcus lentus*	100%	99%
135	BCSA_2	*Staphylococcus lentus*	100%	99%
136	BCSA_3	*Staphylococcus cohnii*	100%	99%
137	BCSA_4	*Staphylococcus nepalensis*	99%	99%
138	BCSA_5	*Staphylococcus cohnii*	100%	100%
139	BCSA_6	*Staphylococcus saprophyticus*	100%	99%
140	BCSA_7	*Staphylococcus saprophyticus*	99%	99%

141	BCSA_8	*Staphylococcus lentus*	100%	99%
142	BCSA_9	*Staphylococcus nepalensis*	100%	99%
143	BCSA_11	*Staphylococcus aureus*	99%	99%
144	BCSA_12	*Staphylococcus cohnii*	99%	98%
145	BCSA_14	*Staphylococcus nepalensis*	99%	99%
146	BCSA_16	*Staphylococcus cohnii*	99%	99%
147	BCSA_17	*Staphylococcus saprophyticus*	100%	99%
148	BCSA_18	*Staphylococcus saprophyticus*	99%	99%
149	BCSA_19	*Staphylococcus nepalensis*	100%	99%
150	BCSA_20	*Staphylococcus nepalensis*	99%	99%
151	BCSA_21	*Staphylococcus nepalensis*	100%	99%
152	CGSA_1	*Staphylococcus sciuri*	99%	99%
153	CGSA_2	*Staphylococcus sciuri*	99%	99%
154	CGSA_3	*Staphylococcus sciuri*	99%	99%
155	CGSA_4	*Staphylococcus sciuri*	99%	99%
156	CGSA_5	*Staphylococcus sciuri*	99%	99%
157	CGSA_7	*Staphylococcus sciuri*	100%	99%
158	CGSA_9	*Staphylococcus sciuri*	99%	99%
159	CGSA_11	*Citrobacter koseri*	99%	99%
160	CGSA_12	*Staphylococcus sciuri*	100%	99%
161	CGSA_13	*Staphylococcus sciuri*	100%	99%
162	CGSA_16	*Staphylococcus sciuri*	99%	99%
163	CGSA_17	*Staphylococcus sciuri*	99%	99%
164	CGSA_18	*Staphylococcus sciuri*	99%	99%
165	CGSA_19	*Staphylococcus sciuri*	99%	99%
166	CGSA_20	*Staphylococcus sciuri*	100%	99%
167	CGSA_21	*Staphylococcus sciuri*	99%	99%
168	G2_LA1	*Myroides phaeus*	99%	96%
169	G2_LA2	*Myroides phaeus*	100%	96%
170	G2_LA4	*Acinetobacter sp.*	99%	98%
171	G2_LA5	*Acinetobacter sp.*	99%	98%
172	G2_LA6	*Acinetobacter gandensis*	99%	98%
173	G2_LA7	*Acinetobacter bouvetii*	99%	98%
174	G2_LA8	*Acinetobacter sp.*	99%	99%
175	G2_LA9	*Comamonas sp.*	99%	99%
176	G2_LA10	*Acinetobacter tjernbergiae*	100%	99%
177	G2_LA11	*Kurthia gibsonii*	100%	99%
178	G2_LA12	*Acinetobacter gandensis*	99%	98%
179	G2_LA15	*Kurthia gibsonii*	99%	99%
180	G2_LA16	*Kurthia gibsonii*	100%	99%
181	G2_LA17	*Acinetobacter sp.*	100%	98%
182	G2_LA18	*Enterobacter sp.*	100%	99%
183	G2_LA19	*Enterobacter sp.*	100%	100%
184	G2_LA20	*Kurthia gibsonii*	99%	99%
185	G2_LA21	*Bacillus pumilus*	100%	99%
186	G3_LA2	*Escherichia coli*	100%	99%
187	G3_LA3	*Escherichia coli*	100%	100%

188	G3_LA4	*Escherichia coli*	100%	100%
189	G3_LA5	*Kurthia gibsonii*	100%	99%
190	G3_LA6	*Escherichia coli*	100%	100%
191	G3_LA7	*Escherichia coli*	100%	100%
192	G3_LA8	*Escherichia coli*	100%	100%
193	G3_LA9	*Escherichia coli*	100%	100%
194	G3_LA10	*Escherichia coli*	100%	100%
195	G3_LA11	*Escherichia coli*	100%	99%
196	G3_LA12	*Escherichia coli*	100%	100%
197	G3_LA13	*Escherichia coli*	100%	100%
198	G3_LA14	*Kurthia gibsonii*	100%	99%
199	G3_LA15	*Kurthia gibsonii*	100%	99%
200	G3_LA17	*Providencia rettgeri*	100%	99%
201	G3_LA18	*Escherichia coli*	100%	100%
202	G3_LA19	*Escherichia coli*	100%	100%
203	G3_LA20	*Escherichia coli*	100%	100%
204	G3_LA21	*Escherichia coli*	100%	100%
205	G6_LA1	*Enterococcus hirae*	100%	99%
206	G6_LA2	*Enterococcus hirae*	100%	99%
207	G6_LA3	*Enterococcus hirae*	100%	99%
208	G6_LA4	*Enterococcus hirae*	100%	99%
209	G6_LA5	*Enterococcus hirae*	100%	99%
210	G6_LA6	*Enterococcus hirae*	100%	99%
211	G6_LA7	*Enterococcus hirae*	100%	99%
212	G6_LA8	*Enterococcus hirae*	100%	99%
213	G6_LA9	*Enterococcus hirae*	100%	99%
214	G6_LA10	*Enterococcus hirae*	100%	99%
215	G6_LA11	*Enterococcus hirae*	100%	99%
216	G6_LA12	*Enterococcus hirae*	100%	99%
217	G6_LA13	*Enterococcus hirae*	100%	99%
218	G6_LA14	*Enterococcus hirae*	100%	99%
219	G6_LA15	*Enterococcus hirae*	100%	99%
220	G6_LA16	*Weissella confusa*	99%	99%
221	G6_LA17	*Enterococcus hirae*	100%	99%
222	G6_LA18	*Enterococcus hirae*	100%	99%
223	G6_LA21	*Enterococcus hirae*	100%	99%
224	G7_LA1	*Staphylococcus sciuri*	100%	99%
225	G7_LA2	*Enterobacter cloacae*	100%	99%
226	G7_LA3	*Enterobacter cloacae*	100%	99%
227	G7_LA4	*Enterobacter cloacae*	100%	99%
228	G7_LA5	*Acinetobacter bouvetii*	99%	97%
229	G7_LA6	*Enterobacter cloacae*	100%	99%
230	G7_LA7	*Enterobacter cloacae*	100%	99%
231	G7_LA9	*Enterobacter cloacae*	100%	99%
232	G7_LA10	*Arthrobacter sp.*	100%	99%
233	G7_LA11	*Enterobacter cloacae*	100%	99%
234	G7_LA13	*Enterobacter cloacae*	100%	99%

235	G7_LA14	*Arthrobacter protophormiae*	100%	99%
236	G7_LA15	*Staphylococcus cohnii*	99%	99%
237	G7_LA16	*Arthrobacter sp.*	100%	99%
238	G7_LA17	*Enterobacter cloacae*	100%	99%
239	G7_LA18	*Enterococcus faecalis*	100%	99%
240	G7_LA19	*Escherichia coli*	100%	99%
241	G7_LA20	*Citrobacter farmeri*	100%	99%
242	G7_LA21	*Enterobacter cloacae*	100%	99%
243	G8_LA1	*Staphylococcus lentus*	99%	99%
244	G8_LA2	*Escherichia coli*	100%	99%
245	G8_LA3	*Escherichia coli*	100%	99%
246	G8_LA4	*Enterobacter sp.*	100%	99%
247	G8_LA5	*Klebsiella oxytoca*	99%	99%
248	G8_LA6	*Escherichia coli*	99%	99%
249	G8_LA7	*Escherichia coli*	99%	99%
250	G8_LA8	*Escherichia coli*	100%	99%
251	G8_LA11	*Escherichia coli*	100%	99%
252	G8_LA12	*Enterobacter cloacae*	100%	99%
253	G8_LA13	*Enterobacter sp.*	99%	99%
254	G8_LA14	*Enterobacter cloacae*	100%	99%
255	G9_LA1	*Enterococcus sp.*	100%	99%
256	G9_LA2	*Enterococcus sp.*	100%	99%
257	G9_LA4	*Yokenella regensburgei*	100%	100%
258	G9_LA5	*Klebsiella sp.*	100%	99%
259	G9_LA6	*Citrobacter koseri*	100%	99%
260	G9_LA7	*Citrobacter koseri*	100%	99%
261	G9_LA8	*Klebsiella sp.*	100%	99%
262	G9_LA9	*Citrobacter koseri*	99%	99%
263	G9_LA10	*Citrobacter koseri*	100%	99%
264	G9_LA13	*Enterococcus sp.*	100%	99%
265	G9_LA15	*Enterobacter cloacae*	100%	99%
266	G15_LA1	*Comamonas testosteroni*	100%	98%
267	G15_LA2	*Sphingobacterium sp.*	100%	99%
268	G15_LA3	*Comamonas sp.*	100%	99%
269	G15_LA4	*Enterococcus sp.*	100%	100%
270	G15_LA5	*Enterococcus thailandicus*	100%	99%
271	G15_LA6	*Enterococcus faecalis*	100%	100%
272	G15_LA9	*Escherichia coli*	100%	100%
273	G15_LA11	*Escherichia coli*	100%	100%
274	G15_LA12	*Acinetobacter haemolyticus*	100%	99%
275	G15_LA13	*Sphingobacterium sp.*	100%	99%
276	G15_LA15	*Enterococcus sp.*	100%	99%
277	G15_LA16	*Enterococcus sp.*	100%	100%
278	G15_LA18	*Wautersiella falsenii*	100%	99%
279	G15_LA20	*Enterobacter cloacae*	100%	99%
280	G15_LA21	*Brevibacterium iodinum*	99%	99%
281	G15B_LA22	*Sphingobacterium sp.*	100%	97%

282	G15B_LA23	*Enterococcus sp.*	100%	100%
283	G15B_LA25	*Comamonas sp.*	100%	96%
284	G15B_LA27	*Pseudomonas sp.*	100%	99%
285	G15B_LA28	*Pseudomonas sp.*	100%	99%
286	G15B_LA29	*Comamonas sp.*	100%	99%
287	G15B_LA30	*Sphingobacterium sp.*	100%	99%
288	G15B_LA31	*Klebsiella pneumoniae*	100%	99%
289	G15B_LA32	*Brevibacterium oceani*	100%	98%
290	G15B_LA33	*Brevibacterium oceani*	98%	99%
291	G17_LA1	*Enterococcus faecalis*	100%	99%
292	G17_LA2	*Weissella confusa*	100%	100%
293	G17_LA3	*Enterococcus faecalis*	100%	99%
294	G17_LA4	*Serratia marcescens*	100%	99%
295	G17_LA5	*Serratia sp.*	100%	100%
296	G17_LA6	*Weissella confusa*	100%	99%
297	G17_LA7	*Staphylococcus lentus*	100%	99%
298	G17_LA8	*Serratia sp.*	100%	100%
299	G17_LA11	*Citrobacter koseri*	100%	99%
300	G17_LA14	*Serratia sp.*	100%	99%
301	G17_LA15	*Tatumella ptyseos*	100%	99%
302	G17_LA16	*Klebsiella sp.*	100%	99%
303	G17_LA17	*Serratia sp.*	100%	99%
304	G17_LA18	*Serratia sp.*	100%	100%
305	G17_LA19	*Serratia sp.*	100%	100%
306	G17_LA20	*Serratia sp.*	100%	100%
307	G17_LA21	*Serratia sp.*	100%	100%
308	G19_LA1	*Enterobacter ludwigii*	100%	99%
309	G19_LA2	*Kurthia gibsonii*	100%	99%
310	G19_LA3	*Acinetobacter haemolyticus*	100%	99%
311	G19_LA4	*Acinetobacter sp.*	100%	99%
312	G19_LA6	*Myroides odoratimimus*	100%	99%
313	G19_LA7	*Serratia sp.*	100%	99%
314	G19_LA8	*Staphylococcus sciuri*	100%	99%
315	G19_LA9	*Enterobacter ludwigii*	99%	99%
316	G19_LA10	*Serratia marcescens*	100%	99%
317	G19_LA11	*Myroides odoratus*	100%	99%
318	G19_LA12	*Kluyvera ascorbata*	100%	99%
319	G19_LA13	*Enterobacter ludwigii*	99%	99%
320	G19_LA14	*Myroides marinus*	100%	99%
321	G19_LA16	*Kurthia gibsonii*	100%	99%
322	G19_LA17	*Enterococcus faecalis*	100%	99%
323	G19_LA18	*Serratia sp.*	100%	99%
324	G19_LA21	*Serratia rubidaea*	99%	99%
325	G20_LA1	*Weissella confusa*	100%	99%
326	G20_LA2	*Weissella confusa*	100%	99%
327	G20_LA3	*Weissella confusa*	99%	99%
328	G20_LA4	*Weissella confusa*	100%	99%

329	G20_LA5	*Weissella confusa*	100%	99%
330	G20_LA6	*Weissella confusa*	99%	99%
331	G20_LA7	*Weissella confusa*	100%	99%
332	G20_LA8	*Weissella confusa*	100%	99%
333	G20_LA9	*Weissella confusa*	99%	99%
334	G20_LA11	*Weissella confusa*	100%	99%
335	G20_LA13	*Weissella confusa*	99%	99%
336	G20_LA14	*Lactococcus lactis*	100%	99%
337	G20_LA15	*Weissella confusa*	100%	99%
338	G20_LA17	*Weissella confusa*	100%	99%
339	G20_LA20	*Weissella confusa*	100%	99%
340	G20_LA21	*Weissella confusa*	99%	99%
341	G20B_LA22	*Weissella confusa*	100%	99%
342	G20B_LA23	*Microbacterium sp.*	99%	99%
343	G20B_LA25	*Weissella confusa*	99%	99%
344	G20B_LA26	*Microbacterium sp.*	99%	99%
345	G20B_LA28	*Microbacterium sp.*	100%	99%
346	G20B_LA29	*Microbacterium sp.*	100%	99%
347	G20B_LA30	*Weissella confusa*	100%	99%
348	G20B_LA31	*Weissella confusa*	100%	99%
349	G20B_LA32	*Weissella confusa*	99%	99%
350	G20B_LA33	*Weissella confusa*	100%	99%
351	G20B_LA34	*Weissella confusa*	100%	99%
352	G20B_LA35	*Weissella confusa*	100%	99%
353	G20B_LA38	*Microbacterium sp.*	99%	99%
354	G20B_LA39	*Microbacterium sp.*	100%	99%
355	G20B_LA40	*Weissella confusa*	100%	99%
356	G20B_LA41	*Lactococcus lactis*	100%	99%
357	G20B_LA42	*Weissella confusa*	100%	99%
358	G21_LA2	*Escherichia coli*	100%	100%
359	G21_LA3	*Escherichia coli*	100%	99%
360	G21_LA4	*Escherichia coli*	100%	99%
361	G21_LA5	*Klebsiella sp.*	100%	99%
362	G21_LA7	*Escherichia coli*	100%	100%
363	G21_LA8	*Escherichia coli*	100%	99%
364	G21_LA9	*Escherichia coli*	100%	99%
365	G21_LA11	*Escherichia coli*	100%	99%
366	G21_LA12	*Escherichia coli*	100%	99%
367	G21_LA13	*Escherichia coli*	100%	99%
368	G21_LA15	*Escherichia fergusonii*	99%	99%
369	G21_LA16	*Escherichia fergusonii*	100%	99%
370	G21_LA17	*Escherichia coli*	100%	100%
371	G21_LA18	*Escherichia coli*	100%	99%
372	G21_LA19	*Escherichia coli*	100%	100%
373	G21_LA20	*Escherichia coli*	100%	99%
374	G21_LA21	*Escherichia coli*	100%	99%
375	G23_LA7	*Enterobacter cloacae*	100%	99%

376	G23_LA8	*Citrobacter koseri*	99%	99%
377	G23_LA9	*Citrobacter koseri*	100%	99%
378	G23_LA10	*Citrobacter koseri*	99%	99%
379	G23_LA11	*Staphylococcus saprophyticus*	100%	99%
380	G23_LA12	*Citrobacter freundii*	100%	99%
381	G23_LA13	*Citrobacter koseri*	99%	99%
382	G23_LA14	*Yokenella regensburgei*	99%	100%
383	G23_LA15	*Citrobacter freundii*	100%	99%
384	G23_LA16	*Enterobacter sp.*	100%	99%
385	G23_LA17	*Citrobacter koseri*	100%	99%
386	G23_LA19	*Citrobacter koseri*	100%	99%
387	G23_LA20	*Citrobacter sp.*	100%	99%
388	G23_LA21	*Klebsiella sp.*	100%	99%
389	G25_LA1	*Staphylococcus lentus*	100%	99%
390	G25_LA2	*Staphylococcus lentus*	100%	99%
391	G25_LA3	*Bacillus altitudinis*	99%	99%
392	G25_LA4	*Enterococcus hirae*	100%	99%
393	G25_LA6	*Bacillus cereus*	100%	99%
394	G25_LA7	*Bacillus megaterium*	99%	99%
395	G25_LA8	*Staphylococcus lentus*	99%	99%
396	G25_LA9	*Staphylococcus lentus*	100%	99%
397	G25_LA10	*Staphylococcus lentus*	100%	99%
398	G25_LA12	*Enterococcus faecalis*	100%	99%
399	G25_LA13	*Staphylococcus cohnii*	99%	99%
400	G25_LA14	*Staphylococcus cohnii*	100%	99%
401	G25_LA15	*Staphylococcus lentus*	99%	99%
402	G25_LA16	*Bacillus sp.*	99%	99%
403	G25_LA17	*Staphylococcus lentus*	99%	99%
404	G25_LA18	*Staphylococcus lentus*	100%	99%
405	G25_LA20	*Staphylococcus saprophyticus*	100%	99%
406	G25_LA21	*Staphylococcus lentus*	99%	99%
407	G25B_LA22	*Enterococcus hirae*	100%	99%
408	G25B_LA23	*Staphylococcus lentus*	99%	99%
409	G25B_LA25	*Staphylococcus lentus*	99%	99%
410	G25B_LA26	*Staphylococcus sp.*	100%	99%
411	G26_LA3	*Enterobacter cloacae*	100%	100%
412	G26_LA4	*Enterobacter cloacae*	100%	99%
413	G26_LA5	*Enterobacter cloacae*	100%	99%
414	G26_LA6	*Enterobacter cloacae*	100%	99%
415	G26_LA7	*Enterobacter cloacae*	100%	100%
416	G26_LA8	*Enterobacter cloacae*	100%	100%
417	G26_LA9	*Enterobacter cloacae*	100%	99%
418	G26_LA11	*Enterobacter cloacae*	100%	99%
419	G26_LA12	*Enterobacter cloacae*	100%	100%
420	G26_LA13	*Enterobacter cloacae*	100%	100%
421	G26_LA14	*Enterobacter cloacae*	100%	100%
422	G26_LA15	*Enterobacter cloacae*	100%	100%

423	G26_LA16	*Enterobacter cloacae*	100%	100%
424	G26_LA17	*Enterobacter cloacae*	100%	100%
425	G26_LA18	*Enterobacter cloacae*	100%	100%
426	G26_LA21	*Enterobacter cloacae*	100%	100%
427	G28_LA1	*Enterococcus faecalis*	100%	99%
428	G28_LA2	*Lactococcus lactis*	100%	99%
429	G28_LA3	*Lactococcus lactis*	99%	99%
430	G28_LA4	*Enterococcus faecalis*	100%	99%
431	G28_LA7	*Enterococcus faecalis*	100%	99%
432	G28_LA9	*Enterococcus faecalis*	100%	99%
433	G28_LA11	*Citrobacter freundii*	100%	99%
434	G28_LA12	*Enterococcus sp.*	99%	99%
435	G28_LA13	*Enterococcus faecalis*	100%	99%
436	G28_LA15	*Lactococcus lactis*	99%	99%
437	G28_LA17	*Enterococcus sp.*	100%	99%
438	G28_LA19	*Lactococcus lactis*	100%	99%
439	G28_LA20	*Escherichia coli*	100%	99%
440	G28_LA21	*Enterococcus saccharolyticus*	99%	99%
441	G28B_LA22	*Enterococcus hirae*	100%	99%
442	G28B_LA23	*Enterococcus hirae*	99%	99%
443	G28B_LA24	*Enterococcus faecalis*	99%	99%
444	G28B_LA25	*Enterococcus sp.*	99%	99%
445	G28B_LA26	*Enterococcus faecalis*	100%	99%
446	G28B_LA27	*Enterococcus faecalis*	100%	99%
447	G28B_LA28	*Enterococcus saccharolyticus*	99%	99%
448	G28B_LA29	*Enterococcus sp.*	100%	99%
449	G28B_LA30	*Enterococcus faecalis*	100%	99%
450	G28B_LA31	*Enterococcus sp.*	99%	99%
451	G28B_LA32	*Enterococcus hirae*	100%	99%
452	G28B_LA33	*Enterococcus hirae*	100%	99%
453	G28B_LA34	*Comamonas sp.*	100%	99%
454	G28B_LA37	*Enterobacter cancerogenus*	100%	99%
455	G28B_LA39	*Enterococcus hirae*	99%	99%
456	G28B_LA40	*Escherichia coli*	100%	99%
457	G28B_LA41	*Enterococcus hirae*	99%	99%
458	G28B_LA42	*Enterococcus hirae*	100%	99%
459	G30_LA1	*Lactococcus lactis*	100%	99%
460	G30_LA2	*Enterococcus faecalis*	99%	100%
461	G30_LA3	*Lactococcus lactis*	99%	99%
462	G30_LA4	*Lactococcus lactis*	100%	99%
463	G30_LA5	*Lactococcus lactis*	100%	99%
464	G30_LA7	*Enterococcus hirae*	100%	100%
465	G30_LA8	*Lactococcus lactis*	100%	99%
466	G30_LA9	*Lactococcus lactis*	100%	99%
467	G30_LA10	*Lactococcus lactis*	100%	99%
468	G30_LA12	*Enterococcus faecalis*	100%	99%
469	G30_LA13	*Enterococcus faecalis*	100%	99%

470	G30_LA14	*Lactococcus lactis*	100%	99%
471	G30_LA15	*Lactococcus lactis*	100%	99%
472	G30_LA16	*Lactococcus lactis*	100%	99%
473	G30_LA17	*Enterobacter cloacae*	99%	99%
474	G30_LA18	*Staphylococcus sciuri*	99%	99%
475	G30_LA19	*Staphylococcus sciuri*	99%	99%
476	G30_LA20	*Lactococcus lactis*	100%	99%
477	G30_LA21	*Lactococcus lactis*	99%	99%
478	G30B_LA22	*Enterococcus faecalis*	100%	99%
479	G30B_LA23	*Lactococcus lactis*	100%	99%
480	G30B_LA24	*Enterobacter cloacae*	100%	99%
481	G30B_LA25	*Staphylococcus sp.*	100%	99%
482	G30B_LA26	*Staphylococcus equorum*	99%	99%
483	G30B_LA28	*Staphylococcus saprophyticus*	100%	100%
484	G30B_LA29	*Vibrio sp.*	100%	95%
485	G30B_LA31	*Koukoulia aurantiaca*	100%	99%
486	G30B_LA32	*Bacillus sp.*	99%	99%
487	G30B_LA33	*Enterococcus hirae*	99%	99%
488	G30B_LA37	*Enterococcus hirae*	100%	99%
489	G30B_LA38	*Lactococcus lactis*	100%	99%
490	G30B_LA39	*Staphylococcus saprophyticus*	100%	99%
491	G30B_LA40	*Staphylococcus saprophyticus*	100%	99%
492	G30B_LA41	*Serratia marcescens*	100%	99%
493	G31_LA1	*Serratia marcescens*	100%	99%
494	G31_LA2	*Citrobacter koseri*	100%	99%
495	G31_LA3	*Citrobacter koseri*	100%	99%
496	G31_LA4	*Citrobacter freundii*	100%	99%
497	G31_LA5	*Enterobacter sp.*	100%	99%
498	G31_LA7	*Enterococcus hirae*	99%	99%
499	G31_LA9	*Klebsiella variicola*	100%	99%
500	G31_LA11	*Citrobacter koseri*	100%	99%
501	G31_LA13	*Lactococcus lactis*	100%	99%
502	G31_LA14	*Lactococcus lactis*	100%	99%
503	G31_LA16	*Enterococcus hirae*	100%	100%
504	G31_LA17	*Klebsiella variicola*	100%	99%
505	G31_LA18	*Citrobacter koseri*	100%	99%
506	G31_LA19	*Citrobacter murliniae*	89%	99%
507	G31_LA20	*Citrobacter freundii*	100%	99%
508	G31_LA21	*Yokenella regensburgei*	99%	99%
509	G31B_LA22	*Enterococcus hirae*	99%	99%
510	G31B_LA23	*Enterococcus hirae*	100%	99%
511	G31B_LA24	*Enterococcus hirae*	100%	99%
512	G31B_LA27	*Enterococcus hirae*	100%	99%
513	G31B_LA28	*Weissella confusa*	100%	100%
514	G31B_LA29	*Enterococcus hirae*	100%	99%
515	G31B_LA30	*Citrobacter freundii*	99%	99%
516	G31B_LA31	*Weissella confusa*	100%	99%

517	G31B_LA33	*Weissella confusa*	100%	100%
518	G31B_LA34	*Citrobacter koseri*	100%	99%
519	G31B_LA35	*Enterococcus hirae*	100%	99%
520	G31B_LA36	*Citrobacter freundii*	99%	99%
521	G31B_LA37	*Weissella confusa*	100%	100%
522	G31B_LA38	*Citrobacter sp.*	100%	99%
523	G31B_LA40	*Enterococcus hirae*	100%	99%
524	G31B_LA41	*Enterobacter ludwigii*	100%	99%
525	BCS_LA1	*Staphylococcus lentus*	100%	99%
526	BCS_LA2	*Staphylococcus cohnii*	100%	99%
527	BCS_LA3	*Staphylococcus lentus*	100%	99%
528	BCS_LA4	*Staphylococcus cohnii*	100%	100%
529	BCS_LA5	*Staphylococcus nepalensis*	100%	100%
530	BCS_LA6	*Staphylococcus lentus*	100%	99%
531	BCS_LA7	*Staphylococcus cohnii*	100%	100%
532	BCS_LA8	*Staphylococcus lentus*	100%	99%
533	BCS_LA9	*Staphylococcus saprophyticus*	100%	100%
534	BCS_LA10	*Staphylococcus lentus*	100%	99%
535	BCS_LA11	*Staphylococcus saprophyticus*	100%	100%
536	BCS_LA12	*Staphylococcus lentus*	100%	99%
537	BCS_LA13	*Staphylococcus aureus*	100%	100%
538	BCS_LA14	*Staphylococcus lentus*	100%	99%
539	BCS_LA15	*Staphylococcus aureus*	100%	100%
540	BCS_LA16	*Staphylococcus lentus*	100%	99%
541	BCS_LA17	*Staphylococcus cohnii*	100%	100%
542	BCS_LA20	*Staphylococcus cohnii*	100%	100%
543	CGS_LA1	*Staphylococcus sciuri*	100%	100%
544	CGS_LA2	*Staphylococcus sciuri*	100%	100%
545	CGS_LA3	*Staphylococcus sciuri*	100%	99%
546	CGS_LA4	*Staphylococcus sciuri*	100%	99%
547	CGS_LA5	*Enterobacter ludwigii*	100%	99%
548	CGS_LA6	*Staphylococcus saprophyticus*	100%	100%
549	CGS_LA7	*Citrobacter koseri*	99%	99%
550	CGS_LA8	*Staphylococcus sciuri*	100%	99%
551	CGS_LA9	*Staphylococcus sciuri*	100%	99%
552	CGS_LA10	*Citrobacter koseri*	100%	99%
553	CGS_LA11	*Enterobacter ludwigii*	100%	99%
554	CGS_LA12	*Staphylococcus sciuri*	100%	99%
555	CGS_LA13	*Staphylococcus sciuri*	99%	100%
556	CGS_LA14	*Staphylococcus lentus*	100%	99%
557	CGS_LA15	*Staphylococcus sciuri*	100%	99%
558	CGS_LA16	*Staphylococcus sciuri*	100%	99%
559	CGS_LA17	*Staphylococcus sciuri*	100%	100%
560	CGS_LA18	*Staphylococcus saprophyticus*	100%	99%
561	CGS_LA19	*Staphylococcus sciuri*	100%	100%
562	CGS_LA21	*Enterobacter sp.*	100%	99%
563	CW_LA1	*Klebsiella oxytoca*	100%	99%

564	CW_LA2	*Klebsiella oxytoca*	100%	99%
565	CW_LA3	*Klebsiella oxytoca*	100%	99%
566	CW_LA5	*Klebsiella sp.*	100%	99%
567	CW_LA7	*Cedecea davisae*	100%	99%
568	CW_LA8	*Enterobacter sp.*	100%	99%
569	CW_LA9	*Pantoea agglomerans*	99%	99%
570	CW_LA10	*Klebsiella sp.*	100%	99%
571	CW_LA11	*Klebsiella sp.*	99%	99%
572	CW_LA12	*Klebsiella oxytoca*	100%	99%
573	CW_LA13	*Klebsiella sp.*	100%	99%
574	CW_LA14	*Klebsiella sp.*	99%	99%
575	CW_LA15	*Klebsiella oxytoca*	100%	99%
576	G2_STR1	*Acinetobacter bouvetii*	99	100
577	G2_STR2	*Acinetobacter gandensis*	99%	98%
578	G2_STR5	*Acinetobacter johnsonii*	100	99
579	G2_STR6	*Acinetobacter haemolyticus*	100%	99%
580	G2_STR7	*Acinetobacter bouvetii*	100	98%
581	G2_STR8	*Acinetobacter gandensis*	99%	98%
582	G2_STR9	*Acinetobacter haemolyticus*	100%	99%
583	G3_STR1	*Escherichia coli*	100	100%
584	G3_STR2	*Escherichia coli*	100	100
585	G3_STR3	*Escherichia coli*	100	99
586	G3_STR4	*Escherichia coli*	100	99
587	G3_STR5	*Escherichia coli*	99	100
588	G3_STR6	*Escherichia coli*	100	100
589	G3_STR7	*Escherichia sp.*	98	99
590	G3_STR9	*Klebsiella oxytoca*	100	99
591	G3_STR10	*Escherichia coli*	100%	99%
592	G3_STR11	*Escherichia coli*	99%	100%
593	G3_STR12	*Escherichia coli*	99	100
594	G3_STR13	*Escherichia coli*	100	99
595	G3_STR14	*Escherichia coli*	100%	99%
596	G3_STR15	*Escherichia coli*	100%	100%
597	G3_STR16	*Escherichia coli*	100%	100%
598	G3_STR17	*Klebsiella oxytoca*	100	99
599	G3_STR18	*Escherichia coli*	99%	99
600	G3_STR19	*Escherichia coli*	99%	99%
601	G3_STR20	*Escherichia coli*	100	100
602	G7_STR1	*Citrobacter murliniae*	98	99
603	G7_STR2	*Enterobacter cloacae*	100	99
604	G7_STR3	*Enterobacter cloacae*	98%	99%
605	G7_STR4	*Citrobacter murliniae*	99	99
606	G7_STR5	*Citrobacter murliniae*	98	99
607	G7_STR6	*Citrobacter murliniae*	98	100
608	G7_STR7	*Citrobacter murliniae*	98	99
609	G7_STR8	*Enterobacter cloacae*	99	99
610	G7_STR9	*Enterobacter cloacae*	99%	99%

611	G7_STR10	*Enterobacter cloacae*	100%	99%
612	G7_STR11	*Enterobacter cloacae*	98%	99%
613	G7_STR12	*Citrobacter murliniae*	100	99
614	G7_STR14	*Enterobacter cloacae*	100%	99%
615	G7_STR15	*Acinetobacter johnsonii*	99%	97%
616	G7_STR16	*Enterobacter cloacae*	99%	99%
617	G7_STR17	*Enterobacter cloacae*	100%	99%
618	G7_STR19	*Enterobacter cloacae*	100%	99%
619	G7_STR20	*Citrobacter murliniae*	100	99%
620	G7_STR21	*Citrobacter murliniae*	100	99%
621	G8_STR1	*Enterobacter cloacae*	100	98%
622	G8_STR2	*Citrobacter koseri*	100	99
623	G8_STR3	*Escherichia coli*	100	99
624	G8_STR4	*Escherichia coli*	99	99
625	G8_STR5	*Citrobacter koseri*	99	99
626	G8_STR6	*Escherichia coli*	99	99
627	G8_STR9	*Escherichia coli*	100	99
628	G8_STR10	*Escherichia coli*	100	99
629	G8_STR11	*Citrobacter koseri*	100	99
630	G8_STR12	*Enterobacter asburiae*	100	99
631	G8_STR13	*Escherichia coli*	100	100
632	G8_STR14	*Escherichia coli*	100	100
633	G8_STR15	*Citrobacter koseri*	100	99
634	G8_STR16	*Enterobacter asburiae*	100	100
635	G8_STR17	*Escherichia coli*	100	100
636	G8_STR18	*Enterobacter asburiae*	100	99
637	G8_STR19	*Enterobacter asburiae*	100	99
638	G8_STR20	*Escherichia coli*	100	100
639	G8_STR21	*Enterobacter ludwigii*	100	99
640	G9_STR1	*Klebsiella oxytoca*	99%	99%
641	G9_STR3	*Klebsiella oxytoca*	100%	99%
642	G9_STR5	*Klebsiella oxytoca*	100	99
643	G9_STR8	*Citrobacter koseri*	100	99
644	G9_STR9	*Citrobacter koseri*	100	99
645	G9_STR11	*Citrobacter koseri*	100	99
646	G9_STR12	*Klebsiella oxytoca*	100	99
647	G15_STR2	*Acinetobacter haemolyticus*	100	99
648	G15_STR3	*Enterobacter cloacae*	100	99
649	G15_STR4	*Acinetobacter haemolyticus*	100	99
650	G15_STR5	*Acinetobacter haemolyticus*	100	99
651	G17_STR1	*Serratia ureilytica*	100	99
652	G17_STR2	*Serratia ureilytica*	100	99
653	G17_STR3	*Serratia ureilytica*	100	100
654	G17_STR4	*Serratia marcescens*	100	99
655	G17_STR5	*Serratia marcescens*	100%	99%
656	G17_STR6	*Serratia marcescens*	100	99
657	G17_STR7	*Serratia marcescens*	100	100

658	G17_STR8	*Serratia ureilytica*	100	99
659	G17_STR9	*Serratia marcescens*	100	99
660	G17_ST10	*Serratia ureilytica*	100	100
661	G17_ST11	*Serratia marcescens*	100	100
662	G17_ST12	*Serratia marcescens*	100%	99%
663	G17_ST13	*Serratia ureilytica*	100	99
664	G17_ST14	*Serratia marcescens*	100	99
665	G17_ST16	*Serratia marcescens*	100%	99%
666	G19_STR1	*Klebsiella pneumoniae*	100	99
667	G19_STR2	*Enterobacter cloacae*	100%	99%
668	G19_STR3	*Acinetobacter sp.*	100	99
669	G19_STR4	*Enterobacter cloacae*	100%	99%
670	G19_STR5	*Acinetobacter lwoffii*	100%	99%
671	G19_STR6	*Pseudomonas putida*	100	100
672	G19_STR7	*Acinetobacter johnsonii*	100%	99%
673	G19_STR8	*Enterobacter cancerogenus*	99	99
674	G19_STR9	*Acinetobacter haemolyticus*	100	99
675	G19_STR10	*Acinetobacter haemolyticus*	100	99
676	G19_STR11	*Enterobacter ludwigii*	100	100
677	G19_STR12	*Leclercia adecarboxylata*	100	100
678	G19_STR13	*Acinetobacter haemolyticus*	100	99
679	G19_STR15	*Klebsiella pneumoniae*	100	100
680	G19_STR16	*Enterobacter cloacae*	100%	99%
681	G19_STR17	*Leclercia adecarboxylata*	100	99
682	G19_STR19	*Serratia marcescens*	100	99
683	G19_STR20	*Citrobacter freundii*	100	99
684	G19_STR21	*Klebsiella oxytoca*	100	99
685	G20_STR1	*Paracoccus alcaliphilus*	100	99
686	G20_STR2	*Paracoccus alcaliphilus*	100	99
687	G20_STR3	*Paracoccus alcaliphilus*	100	99
688	G20_STR5	*Paracoccus alcaliphilus*	100	99
689	G20_STR7	*Paracoccus alcaliphilus*	100	99
690	G20_STR8	*Paracoccus alcaliphilus*	100	99
691	G20_STR9	*Paracoccus alcaliphilus*	100	99
692	G20_STR11	*Citrobacter koseri*	100	99
693	G20_STR12	*Brevibacterium epidermidis*	100	99
694	G23_STR1	*Enterobacter asburiae*	100	99
695	G23_STR2	*Klebsiella oxytoca*	99%	99%
696	G23_STR3	*Enterobacter asburiae*	99	99
697	G23_STR4	*Enterobacter asburiae*	99%	99%
698	G23_STR5	*Enterobacter asburiae*	100	99
699	G23_STR6	*Citrobacter koseri*	99	99
700	G23_STR7	*Klebsiella oxytoca*	100%	100%
701	G23_STR8	*Enterobacter asburiae*	100	100
702	G23_STR9	*Enterobacter asburiae*	100	99
703	G23_STR11	*Enterobacter asburiae*	99	99
704	G23_STR12	*Enterobacter asburiae*	100	99

705	G23_STR13	*Enterobacter asburiae*	100	99
706	G23_STR14	*Enterobacter asburiae*	100	100
707	G23_STR15	*Enterobacter asburiae*	100	99
708	G23_STR16	*Enterobacter asburiae*	100	99
709	G23_STR17	*Enterobacter asburiae*	100	99
710	G23_STR18	*Enterobacter asburiae*	100	99
711	G23_STR19	*Enterobacter asburiae*	100	100
712	G23_STR20	*Enterobacter asburiae*	100	99
713	G26_STR1	*Citrobacter freundii*	100	99
714	G26_STR2	*Enterobacter cloacae*	99	99
715	G26_STR3	*Citrobacter freundii*	100	100
716	G26_STR4	*Enterobacter cloacae*	100	100
717	G26_STR5	*Enterobacter cloacae*	100	100
718	G26_STR6	*Enterobacter cloacae*	100	100
719	G26_STR7	*Enterobacter cloacae*	100	100
720	G26_STR8	*Enterobacter cloacae*	99	99
721	G26_STR9	*Enterobacter cloacae*	100	100
722	G26_STR10	*Citrobacter freundii*	100	99
723	G26_STR11	*Enterobacter cloacae*	100	100
724	G26_STR12	*Citrobacter freundii*	100	99
725	G26_STR13	*Enterobacter cloacae*	100	99
726	G26_STR14	*Enterobacter cloacae*	99	99
727	G26_STR15	*Enterobacter cloacae*	100	99
728	G26_STR16	*Enterobacter cloacae*	100	99
729	G26_STR17	*Enterobacter cloacae*	100	100
730	G26_STR18	*Enterobacter cloacae*	100	99
731	G26_STR19	*Enterobacter cloacae*	100	99
732	G26_STR20	*Enterobacter cloacae*	100	99
733	G26_STR21	*Enterobacter cloacae*	100	100
734	G28_STR2	*Enterobacter ludwigii*	100	99
735	G28_STR3	*Acinetobacter haemolyticus*	100	99
736	G28_STR5	*Raoultella ornithinolytica*	100	99
737	G28_STR6	*Escherichia coli*	100	100
738	G28_STR7	*Escherichia coli*	100	99
739	G28_STR9	*Enterobacter asburiae*	100	100
740	G28_STR10	*Klebsiella oxytoca*	100%	99%
741	G28_STR11	*Escherichia coli*	100	99
742	G28_STR12	*Klebsiella oxytoca*	100%	99%
743	G28_STR13	*Enterobacter cloacae*	100%	99%
744	G28_STR15	*Escherichia coli*	100	99
745	G28_STR18	*Raoultella ornithinolytica*	100	99
746	G28_STR19	*Acinetobacter haemolyticus*	100	99
747	G28_STR20	*Escherichia coli*	100	99
748	G28_STR21	*Pseudomonas putida*	100	99
749	G30_STR1	*Escherichia coli*	100	99
750	G30_STR5	*Arthrobacter arilaitensis*	100%	98%
751	G30_STR6	*Enterobacter asburiae*	100	99

752	G30_STR7	*Acinetobacter haemolyticus*	100	99
753	G30_STR11	*Serratia marcescens*	100	99
754	G30_STR16	*Enterobacter ludwigii*	100	99
755	G30_STR17	*Acinetobacter junii*	100	99
756	G31_STR1	*Enterobacter cloacae*	99	99
757	G31_STR2	*Enterobacter sp.*	100%	99%
758	G31_STR5	*Klebsiella variicola*	100	99
759	G31_STR7	*Citrobacter koseri*	99	99
760	G31_STR8	*Enterobacter ludwigii*	100	99
761	G31_STR9	*Enterobacter hormaechei*	100%	99%
762	G31_STR11	*Enterobacter sp.*	100%	99%
763	G31_STR13	*Enterobacter ludwigii*	100	99
764	G31_STR14	*Enterobacter cancerogenus*	100	99
765	G31_STR15	*Klebsiella variicola*	100	99
766	G31_STR16	*Enterobacter asburiae*	99	100
767	G31_STR20	*Klebsiella oxytoca*	100	99
768	CGS_STR2	*Enterobacter cloacae*	100%	100%
769	CGS_STR3	*Enterobacter asburiae*	100	99
770	CGS_STR4	*Enterobacter asburiae*	100	99
771	CGS_STR5	*Enterobacter cloacae*	100%	100%
772	CGS_STR7	*Enterobacter cloacae*	100%	99%
773	CGS_STR10	*Enterobacter asburiae*	100	99
774	CGS_STR12	*Enterobacter asburiae*	100	98
775	CGS_STR13	*Enterobacter cloacae*	100%	99%
776	CGS_STR15	*Enterobacter asburiae*	100	99
777	CGS_STR16	*Enterobacter ludwigii*	100	99
778	CGS_STR17	*Enterobacter cloacae*	100	99
779	CGS_STR18	*Enterobacter cloacae*	100%	100%
780	CGS_STR19	*Enterobacter asburiae*	100	99
781	CGS_STR20	*Citrobacter koseri*	99	99
782	CW_STR_1	*Aquitalea denitrificans*	99%	98%
783	CW_STR_3	*Aquitalea sp.*	100%	99%
784	CW_STR5	*Enterobacter cancerogenus*	100	99
785	CW_STR6	*Enterobacter cloacae*	100	99
786	CW_STR7	*Aquitalea magnusonii*	100	99
787	G2_ZM1	*Lactococcus lactis*	100%	99%
788	G2_ZM2	*Lactococcus lactis*	100%	99%
789	G2_ZM3	*Lactococcus lactis*	100%	99%
790	G2_ZM4	*Acinetobacter haemolyticus*	100%	99%
791	G2_ZM5	*Acinetobacter guillouiae*	99%	98%
792	G2_ZM6	*Acinetobacter guillouiae*	100%	99%
793	G2_ZM7	*Brevibacterium oceani*	100%	99%
794	G3_ZM1	*Escherichia fergusonii*	100%	99%
795	G3_ZM2	*Escherichia fergusonii*	100%	99%
796	G3_ZM3	*Enterobacter soli*	100%	99%
797	G3_ZM4	*Escherichia coli*	100%	99%
798	G3_ZM5	*Escherichia fergusonii*	99%	99%

799	G3_ZM6	*Escherichia coli*	99%	99%
800	G3_ZM7	*Escherichia coli*	99%	99%
801	G3_ZM8	*Escherichia coli*	100%	99%
802	G3_ZM9	*Escherichia fergusonii*	99%	99%
803	G3_ZM10	*Escherichia fergusonii*	100%	99%
804	G3_ZM11	*Escherichia fergusonii*	100%	99%
805	G3_ZM12	*Escherichia fergusonii*	100%	99%
806	G3_ZM13	*Escherichia fergusonii*	100%	99%
807	G3_ZM14	*Acinetobacter haemolyticus*	98%	97%
808	G3_ZM15	*Escherichia coli*	100%	99%
809	G3_ZM16	*Escherichia coli*	98%	99%
810	G3_ZM17	*Escherichia coli*	99%	99%
811	G3_ZM18	*Escherichia coli*	99%	99%
812	G3_ZM19	*Escherichia fergusonii*	100%	99%
813	G3_ZM20	*Escherichia coli*	99%	99%
814	G3_ZM21	*Escherichia fergusonii*	99%	99%
815	G6_ZM1	*Enterococcus hirae*	99%	99%
816	G6_ZM2	*Enterococcus hirae*	99%	99%
817	G6_ZM3	*Enterococcus hirae*	99%	99%
818	G6_ZM4	*Enterococcus hirae*	99%	99%
819	G6_ZM5	*Enterococcus hirae*	99%	99%
820	G6_ZM6	*Enterococcus hirae*	98%	99%
821	G6_ZM7	*Enterococcus hirae*	99%	99%
822	G6_ZM8	*Enterococcus hirae*	100%	100%
823	G6_ZM9	*Enterococcus hirae*	100%	99%
824	G6_ZM11	*Enterococcus hirae*	99%	99%
825	G6_ZM12	*Enterococcus hirae*	99%	99%
826	G6_ZM13	*Enterococcus hirae*	99%	99%
827	G6_ZM14	*Enterococcus hirae*	99%	99%
828	G6_ZM15	*Enterococcus hirae*	99%	99%
829	G6_ZM17	*Enterococcus hirae*	99%	99%
830	G6_ZM18	*Enterococcus hirae*	99%	99%
831	G6_ZM19	*Enterococcus hirae*	99%	99%
832	G7_ZM1	*Arthrobacter protophormiae*	99%	99%
833	G7_ZM2	*Citrobacter murliniae*	98%	99%
834	G7_ZM3	*Arthrobacter soli*	98%	99%
835	G7_ZM4	*Enterobacter ludwigii*	99%	98%
836	G7_ZM5	*Arthrobacter soli*	98%	99%
837	G7_ZM6	*Arthrobacter soli*	98%	99%
838	G7_ZM7	*Enterococcus faecalis*	98%	99%
839	G7_ZM9	*Arthrobacter soli*	99%	99%
840	G7_ZM10	*Enterobacter ludwigii*	99%	99%
841	G7_ZM11	*Aerococcus viridans*	99%	99%
842	G7_ZM12	*Citrobacter freundii*	100%	99%
843	G7_ZM13	*Enterobacter ludwigii*	99%	98%
844	G7_ZM14	*Acinetobacter johnsonii*	99%	97%
845	G7_ZM15	*Aerococcus viridans*	99%	99%

846	G7_ZM17	*Arthrobacter soli*	99%	99%
847	G7_ZM18	*Enterobacter ludwigii*	100%	99%
848	G7_ZM19	*Acinetobacter johnsonii*	100%	97%
849	G7_ZM20	*Enterococcus faecalis*	100%	99%
850	G7_ZM21	*Enterobacter ludwigii*	100%	99%
851	G8_ZM1	*Enterobacter cloacae*	100%	99%
852	G8_ZM2	*Staphylococcus kloosii*	99%	99%
853	G8_ZM3	*Escherichia coli*	99%	99%
854	G8_ZM4	*Escherichia coli*	100%	99%
855	G8_ZM5	*Enterobacter asburiae*	100%	99%
856	G8_ZM6	*Enterobacter cloacae*	100%	99%
857	G8_ZM7	*Escherichia coli*	100%	99%
858	G8_ZM8	*Escherichia coli*	100%	100%
859	G8_ZM9	*Escherichia coli*	100%	99%
860	G8_ZM10	*Escherichia coli*	99%	99%
861	G8_ZM13	*Enterobacter cloacae*	100%	99%
862	G8_ZM14	*Escherichia coli*	100%	99%
863	G8_ZM15	*Escherichia coli*	99%	99%
864	G8_ZM16	*Enterobacter asburiae*	99%	99%
865	G8_ZM17	*Staphylococcus nepalensis*	99%	99%
866	G8_ZM18	*Escherichia coli*	99%	99%
867	G8_ZM20	*Enterobacter asburiae*	100%	99%
868	G9_ZM1	*Enterococcus hirae*	99%	99%
869	G9_ZM2	*Citrobacter koseri*	100%	99%
870	G9_ZM3	*Enterococcus hirae*	99%	99%
871	G9_ZM5	*Citrobacter koseri*	99%	99%
872	G9_ZM6	*Citrobacter koseri*	99%	99%
873	G9_ZM7	*Klebsiella oxytoca*	100%	99%
874	G15_ZM1	*Pseudomonas composti*	99%	99%
875	G15_ZM2	*Enterobacter asburiae*	99%	99%
876	G15_ZM4	*Acinetobacter haemolyticus*	99%	99%
877	G15_ZM5	*Acinetobacter johnsonii*	100%	99%
878	G15_ZM6	*Enterococcus faecalis*	100%	100%
879	G15_ZM7	*Escherichia fergusonii*	99%	99%
880	G15_ZM8	*Citrobacter freundii*	99%	99%
881	G15_ZM9	*Staphylococcus kloosii*	99%	99%
882	G17_ZM2	*Lactococcus lactis*	99%	99%
883	G17_ZM3	*Serratia nematodiphila*	99%	99%
884	G17_ZM4	*Staphylococcus lentus*	99%	99%
885	G17_ZM5	*Serratia nematodiphila*	99%	99%
886	G17_ZM6	*Serratia ureilytica*	100%	99%
887	G17_ZM7	*Lactococcus lactis*	100%	100%
888	G17_ZM8	*Serratia ureilytica*	100%	99%
889	G17_ZM9	*Serratia ureilytica*	100%	100%
890	G17_ZM10	*Serratia marcescens*	100%	100%
891	G17_ZM11	*Serratia ureilytica*	100%	100%
892	G17_ZM12	*Staphylococcus lentus*	99%	99%

893	G19_ZM1	*Enterobacter ludwigii*	100%	99%
894	G19_ZM4	*Kurthia gibsonii*	100%	99%
895	G19_ZM7	*Acinetobacter haemolyticus*	99%	99%
896	G19_ZM8	*Klebsiella variicola*	99%	99%
897	G19_ZM10	*Escherichia coli*	99%	100%
898	G19_ZM11	*Arthrobacter protophormiae*	99%	99%
899	G19_ZM13	*Providencia alcalifaciens*	100%	99%
900	G19_ZM15	*Yersinia massiliensis*	100%	99%
901	G19_ZM16	*Providencia alcalifaciens*	100%	99%
902	G19_ZM17	*Proteus mirabilis*	100%	100%
903	G19_ZM18	*Klebsiella oxytoca*	99%	100%
904	G19_ZM19	*Enterococcus faecalis*	99%	99%
905	G19_ZM20	*Serratia nematodiphila*	100%	99%
906	G20_ZM1	*Brevibacterium epidermidis*	99%	99%
907	G20_ZM2	*Brachybacterium paraconglomeratum*	100%	99%
908	G20_ZM3	*Brachybacterium paraconglomeratum*	100%	99%
909	G20_ZM4	*Serratia marcescens*	100%	99%
910	G20_ZM5	*Lactococcus lactis*	99%	99%
911	G20_ZM6	*Aerococcus viridans*	100%	99%
912	G20_ZM7	*Paracoccus alcaliphilus*	100%	99%
913	G20_ZM8	*Brachybacterium paraconglomeratum*	99%	99%
914	G20_ZM9	*Microbacterium amylolyticum*	98%	98%
915	G20_ZM10	*Brachybacterium paraconglomeratum*	100%	99%
916	G20_ZM11	*Aerococcus urinaeequi*	100%	99%
917	G20_ZM12	*Lactococcus lactis*	100%	99%
918	G20_ZM13	*Brachybacterium paraconglomeratum*	100%	99%
919	G20_ZM14	*Brachybacterium paraconglomeratum*	99%	99%
920	G20_ZM16	*Brachybacterium paraconglomeratum*	99%	99%
921	G20_ZM18	*Brachybacterium paraconglomeratum*	100%	99%
922	G20_ZM19	*Brachybacterium paraconglomeratum*	100%	99%
923	G20_ZM21	*Lactococcus lactis*	100%	99%
924	G21_ZM1	*Escherichia fergusonii*	99%	99%
925	G21_ZM2	*Escherichia coli*	99%	99%
926	G21_ZM3	*Escherichia fergusonii*	99%	99%
927	G21_ZM4	*Escherichia coli*	100%	99%
928	G21_ZM5	*Escherichia fergusonii*	99%	99%
929	G21_ZM6	*Escherichia coli*	100%	99%
930	G21_ZM7	*Escherichia coli*	99%	99%
931	G21_ZM8	*Escherichia coli*	99%	99%
932	G21_ZM9	*Escherichia coli*	99%	99%
933	G23_ZM2	*Citrobacter freundii*	99%	99%
934	G23_ZM3	*Enterobacter asburiae*	99%	99%
935	G23_ZM4	*Citrobacter freundii*	99%	99%
936	G23_ZM5	*Citrobacter freundii*	100%	99%
937	G23_ZM6	*Enterobacter ludwigii*	100%	99%
938	G23_ZM7	*Enterobacter mori*	100%	99%
939	G23_ZM8	*Enterobacter asburiae*	100%	99%

940	G23_ZM9	*Citrobacter koseri*	99%	99%
941	G23_ZM10	*Citrobacter freundii*	99%	99%
942	G23_ZM11	*Citrobacter freundii*	100%	99%
943	G23_ZM12	*Staphylococcus lentus*	100%	100%
944	G23_ZM14	*Citrobacter freundii*	100%	99%
945	G23_ZM15	*Citrobacter koseri*	100%	99%
946	G23_ZM17	*Enterococcus faecalis*	100%	99%
947	G23_ZM18	*Citrobacter freundii*	100%	99%
948	G25_ZM2	*Staphylococcus lentus*	100%	99%
949	G25_ZM3	*Staphylococcus lentus*	99%	99%
950	G25_ZM4	*Staphylococcus lentus*	99%	99%
951	G25_ZM9	*Enterococcus faecalis*	100%	99%
952	G25_ZM10B	*Enterococcus faecalis*	99%	99%
953	G25_ZM11B	*Enterococcus hirae*	99%	99%
954	G25_ZM14	*Staphylococcus cohnii*	99%	99%
955	G25_ZM17B	*Staphylococcus lentus*	99%	99%
956	G25_ZM20B	*Staphylococcus lentus*	99%	98%
957	G25_ZM21B	*Staphylococcus cohnii*	98%	99%
958	G25_ZM22B	*Corynebacterium glutamicum*	99%	97%
959	G25_ZM24B	*Staphylococcus lentus*	100%	99%
960	G26_ZM1	*Enterobacter cloacae*	99%	99%
961	G26_ZM2	*Enterobacter cloacae*	99%	99%
962	G26_ZM3	*Enterobacter cloacae*	99%	99%
963	G26_ZM4	*Enterobacter cloacae*	99%	99%
964	G26_ZM5	*Enterobacter cloacae*	98%	100%
965	G26_ZM6	*Enterobacter cloacae*	99%	99%
966	G26_ZM7	*Enterobacter cloacae*	99%	99%
967	G26_ZM8	*Enterobacter cloacae*	99%	99%
968	G26_ZM9	*Enterobacter cloacae*	99%	99%
969	G26_ZM10	*Enterobacter cloacae*	100%	99%
970	G26_ZM11	*Enterobacter cloacae*	100%	99%
971	G26_ZM12	*Enterobacter cloacae*	99%	99%
972	G26_ZM13	*Enterobacter ludwigii*	99%	99%
973	G26_ZM14	*Enterobacter cloacae*	99%	99%
974	G26_ZM15	*Enterobacter cloacae*	99%	99%
975	G26_ZM16	*Enterobacter ludwigii*	98%	99%
976	G26_ZM17	*Enterobacter cloacae*	99%	99%
977	G26_ZM18	*Enterobacter cloacae*	99%	99%
978	G26_ZM19	*Enterobacter cloacae*	100%	99%
979	G26_ZM20	*Enterobacter cloacae*	100%	99%
980	G26_ZM21	*Enterobacter cloacae*	99%	99%
981	G28_ZM1	*Escherichia coli*	100%	99%
982	G28_ZM3	*Enterococcus faecalis*	99%	99%
983	G28_ZM4	*Enterococcus faecalis*	99%	99%
984	G28_ZM5	*Enterococcus faecalis*	99%	99%
985	G28_ZM7	*Enterococcus casseliflavus*	100%	99%
986	G28_ZM8	*Enterococcus faecalis*	99%	99%

987	G28_ZM9	*Lactococcus lactis*	99%	99%
988	G28_ZM11	*Enterococcus hirae*	99%	99%
989	G28_ZM12	*Lactococcus lactis*	99%	99%
990	G28_ZM13	*Escherichia coli*	99%	99%
991	G28_ZM15	*Lactococcus lactis*	99%	99%
992	G28_ZM16	*Enterococcus faecalis*	100%	100%
993	G28_ZM17	*Enterococcus saccharolyticus*	100%	99%
994	G28_ZM18	*Escherichia coli*	100%	99%
995	G28_ZM19	*Enterococcus hirae*	100%	100%
996	G28_ZM21	*Enterococcus hirae*	100%	99%
997	G30_ZM1	*Enterococcus faecalis*	100%	99%
998	G30_ZM2	*Enterococcus faecalis*	99%	100%
999	G30_ZM3	*Advenella mimigardefordensis*	100%	99%
1000	G30_ZM4	*Lactococcus lactis*	100%	100%
1001	G30_ZM6	*Lactococcus lactis*	100%	99%
1002	G30_ZM7	*Staphylococcus aureus*	100%	99%
1003	G30_ZM8	*Lactococcus lactis*	100%	100%
1004	G30_ZM9	*Lactococcus lactis*	100%	99%
1005	G30_ZM10	*Staphylococcus equorum*	99%	99%
1006	G30_ZM11	*Serratia marcescens*	99%	99%
1007	G30_ZM12	*Staphylococcus equorum*	99%	99%
1008	G30_ZM13	*Lactococcus lactis*	99%	99%
1009	G30_ZM17	*Kurthia huakuii*	100%	99%
1010	G30_ZM18	*Staphylococcus aureus*	99%	99%
1011	G30_ZM19	*Brachybacterium paraconglomeratum*	100%	99%
1012	G30_ZM21	*Staphylococcus equorum*	100%	99%
1013	G31_ZM1	*Klebsiella variicola*	99%	100%
1014	G31_ZM2	*Enterococcus hirae*	100%	99%
1015	G31_ZM4	*Lactococcus lactis*	100%	99%
1016	G31_ZM6	*Enterococcus hirae*	99%	99%
1017	G31_ZM7	*Enterococcus hirae*	100%	99%
1018	G31_ZM8	*Enterococcus hirae*	100%	99%
1019	G31_ZM9	*Enterococcus hirae*	100%	99%
1020	G31_ZM10	*Enterobacter hormaechei*	100%	99%
1021	G31_ZM12	*Enterococcus hirae*	99%	99%
1022	G31_ZM14	*Lactococcus lactis*	99%	99%
1023	G31_ZM16	*Citrobacter freundii*	100%	99%
1024	G31_ZM17	*Enterococcus hirae*	100%	99%
1025	G31_ZM18	*Citrobacter freundii*	99%	99%
1026	G31_ZM20	*Citrobacter koseri*	99%	99%
1027	BCS_ZM2	*Staphylococcus nepalensis*	100%	100%
1028	BCS_ZM3	*Staphylococcus lentus*	100%	99%
1029	BCS_ZM4	*Staphylococcus cohnii*	100%	100%
1030	BCS_ZM5	*Staphylococcus lentus*	100%	99%
1031	BCS_ZM6	*Staphylococcus nepalensis*	99%	100%
1032	BCS_ZM7	*Staphylococcus nepalensis*	100%	100%
1033	BCS_ZM8	*Staphylococcus cohnii*	99%	100%

1034	BCS_ZM9	*Staphylococcus cohnii*	100%	99%
1035	BCS_ZM10	*Staphylococcus lentus*	99%	99%
1036	BCS_ZM11	*Staphylococcus lentus*	99%	99%
1037	BCS_ZM12	*Staphylococcus cohnii*	100%	99%
1038	BCS_ZM13	*Staphylococcus cohnii*	100%	99%
1039	BCS_ZM14	*Staphylococcus lentus*	99%	99%
1040	BCS_ZM15	*Staphylococcus lentus*	100%	99%
1041	BCS_ZM16	*Staphylococcus nepalensis*	100%	100%
1042	BCS_ZM17	*Staphylococcus lentus*	100%	99%
1043	BCS_ZM18	*Staphylococcus lentus*	100%	99%
1044	BCS_ZM19	*Staphylococcus nepalensis*	100%	99%
1045	BCS_ZM20	*Staphylococcus aureus*	100%	99%
1046	BCS_ZM21	*Staphylococcus lentus*	99%	99%
1047	CGS_ZM1	*Staphylococcus saprophyticus*	100%	100%
1048	CGS_ZM2	*Staphylococcus sciuri*	100%	100%
1049	CGS_ZM3	*Staphylococcus lentus*	100%	99%
1050	CGS_ZM4	*Staphylococcus lentus*	99%	99%
1051	CGS_ZM5	*Staphylococcus sciuri*	100%	99%
1052	CGS_ZM6	*Staphylococcus lentus*	99%	99%
1053	CGS_ZM8	*Staphylococcus sciuri*	100%	99%
1054	CGS_ZM9	*Staphylococcus sciuri*	99%	99%
1055	CGS_ZM10	*Staphylococcus sciuri*	100%	99%
1056	CGS_ZM11	*Staphylococcus sciuri*	100%	100%
1057	CGS_ZM12	*Staphylococcus sciuri*	100%	99%
1058	CGS_ZM14	*Staphylococcus lentus*	99%	99%
1059	CGS_ZM15	*Staphylococcus sciuri*	99%	100%
1060	CGS_ZM16	*Staphylococcus sciuri*	100%	99%
1061	CGS_ZM18	*Staphylococcus sciuri*	100%	99%
1062	CGS_ZM19	*Staphylococcus sciuri*	100%	99%
1063	CGS_ZM21	*Staphylococcus sciuri*	100%	99%
1064	CW_ZM1	*Enterococcus faecalis*	100%	99%
1065	CW_ZM3	*Enterococcus faecalis*	99%	99%
