# Supplementary material for: Microbiome analysis reveals the abundance of bacterial pathogens in Rousettus leschenaultii guano
Source: Sci Rep. 2016 Nov 15;6:36948. doi: 10.1038/srep36948 (PMC5109407; doi:10.1038/srep36948)
Supplement: Supplementary Information [file srep36948-s2.pdf]

**Title: Microbiome analysis reveals the abundance of bacterial pathogens in *Rousettus leschenaultii* guano**

Sunil Banskar [1], Shrikant S. Bhute [2], Mangesh V. Suryavanshi [1], Sachin Punekar [3]

Yogesh S. Shouche [1]*

**Author Affiliations:**

[1] Microbial Culture Collection, National Centre for Cell Science, Pune, India.

[2] Savitribai Phule Pune University, Pune, India.

[3] Biosphere, Eshwari, 52/403, Lakshminagar, Parvati, Pune-411009 (Maharashtra), India.

* Correspondence:

**Dr. Yogesh S. Shouche**

Microbial Culture Collection

National Centre for Cell Science,

Central Tower, Sai Trinity Building Garware Circle,

Sutarwadi, Pashan Pune, Maharashtra 411021, India

Fax No: +91-20-25329001

Telephone No: +91-20-25329000

Email: yogesh@nccs.res.in

**Supplementary Table-S1. Supplementary Table-S1.pdf** file attached separately

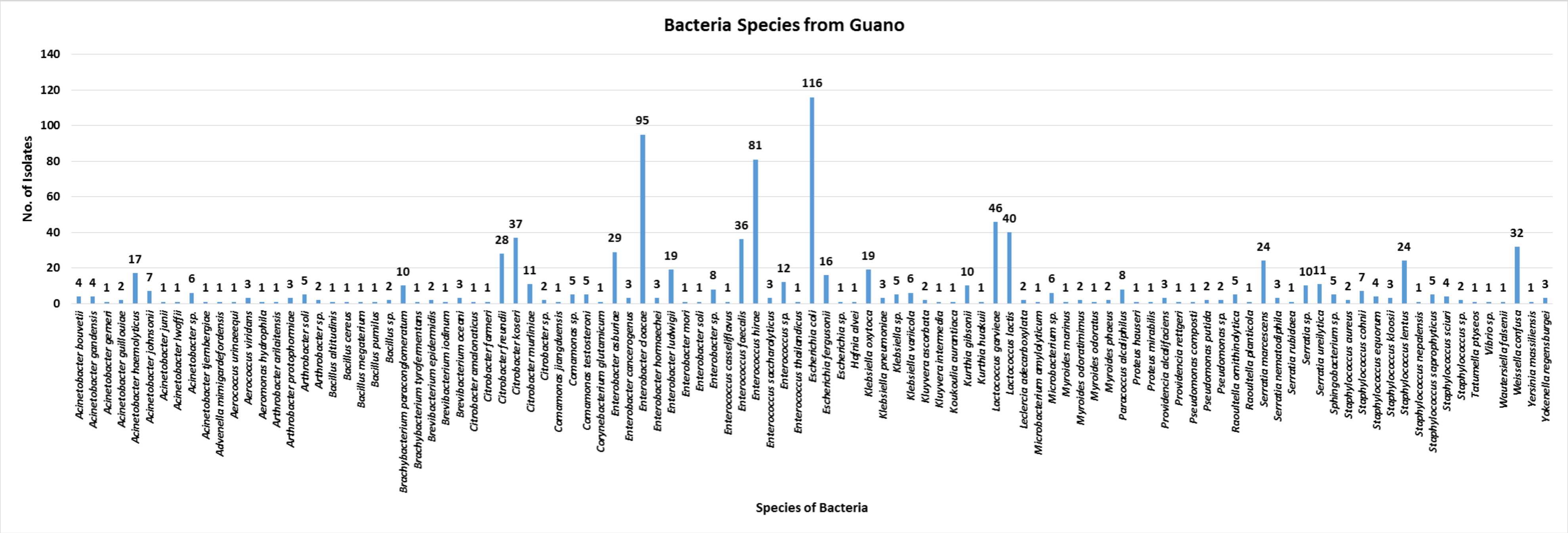


**Supplementary Figure-S2.** The different bacterial species isolated from the bat guano.

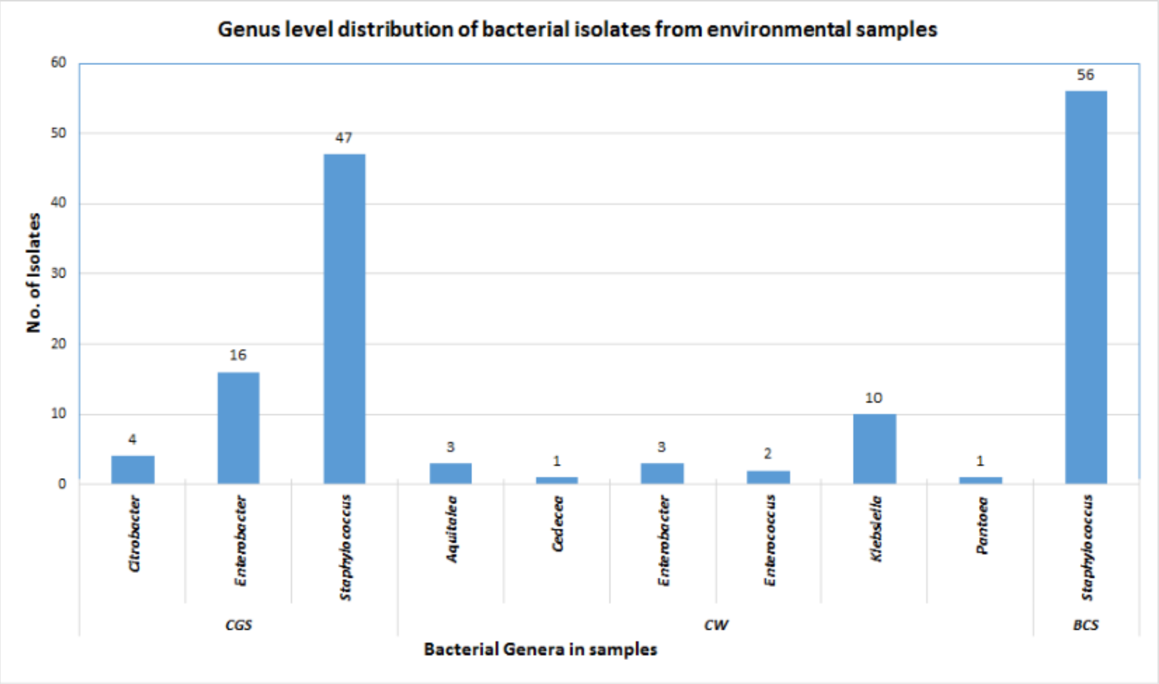


**Supplementary Figure-S3.** The bacterial genera obtained from the cave environment. A single bacterial genus *i.e. Staphylococcus* was obtained from BCS sample.

**Supplementary Table-S4:** The list of primers used for the identification of *E. coli* pathotypes.

| Sr. No. | virulence gene | Primer name | DNA Sequence (5' to 3') | Pathotype | Pathogenity type | Description |
|---|---|---|---|---|---|---|
| 1 | *hlyA* | hly-F | AACAAGGATAAGCACTGTTCTGGCT | ExPEC | extraintestinal (ExPEC) | Alpha-Hemolysin |
| | | hly-R | ACCATATAAGCGGTCATTCCCGTCA | | | |
| 2 | *ibeA* | ibe10-F | AGGCAGGTGTGCGCCGCGTAC | | | Invasion of brain endothelium |
| | | ibe10-R | TGGTGCTCCGGCAAACCATGC | | | |
| 3 | *east1* | east 11a | CCATCAACACAGTATATCCGA | EaggEC | entero-aggregative (EaggEC) | heat-stable enterotoxin |
| | | east 11b | GGTCGCGAGTGACGGCTTTGT | | | |
| 4 | *stx1* | stx1-F | ATAAATCGCCTATCGTTGACTAC | EHEC | enterohemorrhagic (EHEC) | Shiga toxin-1 |
| | | stx1-R | AGAACGCCCACTGAGATCATC | | | |
| 5 | *stx2* | stx2-F | GGCACTGTCTGAAACTGCTCC | | | Shiga toxin-2 |
| | | stx2-R | TCGCCAGTTATCTGACATTCTG | | | |
| 6 | *ipah* | ipaHIII | GTTCCTTGACCGCCTTTCCGATACCGTC | EIEC | enteroinvasive (EIEC), | Invasion Plasmid antigen |
| | | ipaHIV | GCCGGTCAGCCACCCTCTGAGATAC | | | |
| 7 | *estII* | STb-2 | ATCGCATTTCTTCTTGCATC | ETEC | enterotoxigenic (ETEC) | Heat stable toxin-b |
| | | STb-2 | GGGCGCCAAAGCATGCTCC | | | |
| 8 | *estI* | STa1 | TCTTTCCCCTCTTTTAGTCAG | | | Heat stable toxin-a |
| | | STa1 | ACAGGCAGGATTACAACAAAG | | | |

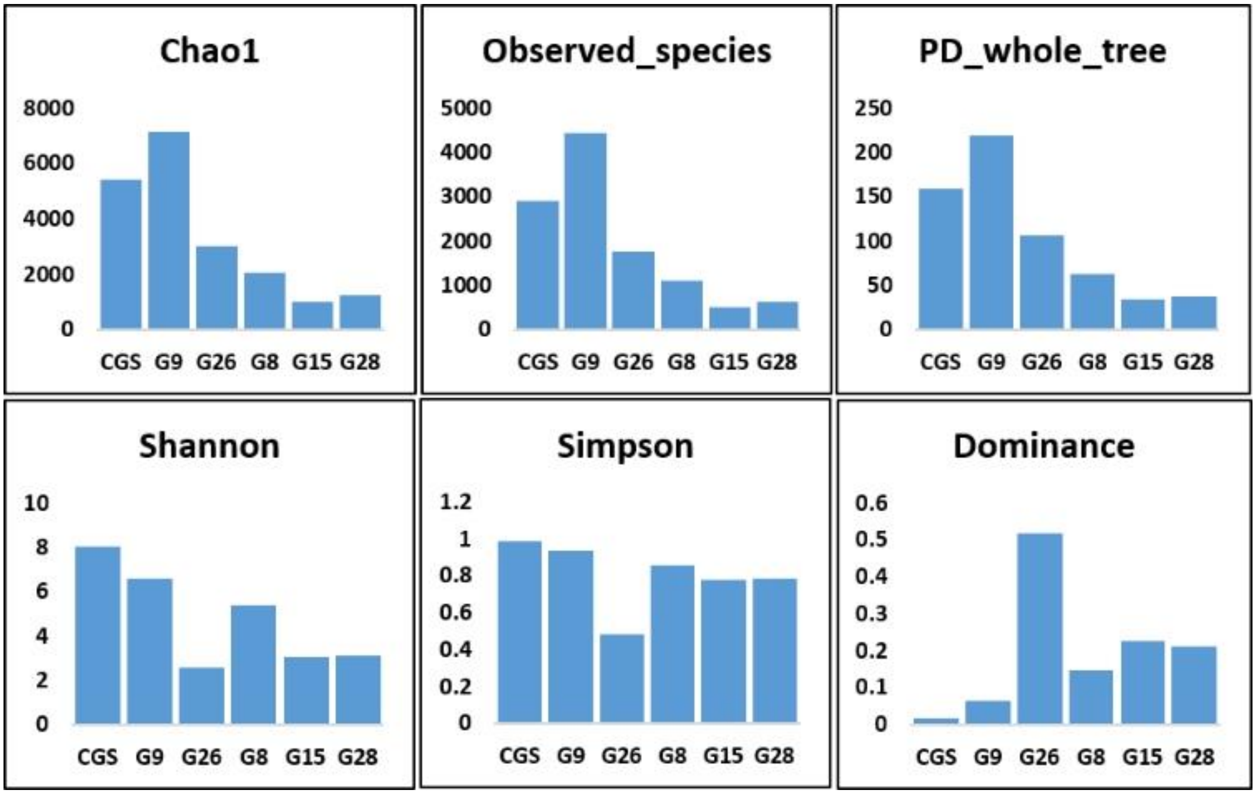


**Supplementary Figure-S5.** The alpha diversity indices showing the diversity of bacterial communities in different guano samples and a composite (decaying) guano *i.e.* CGS.

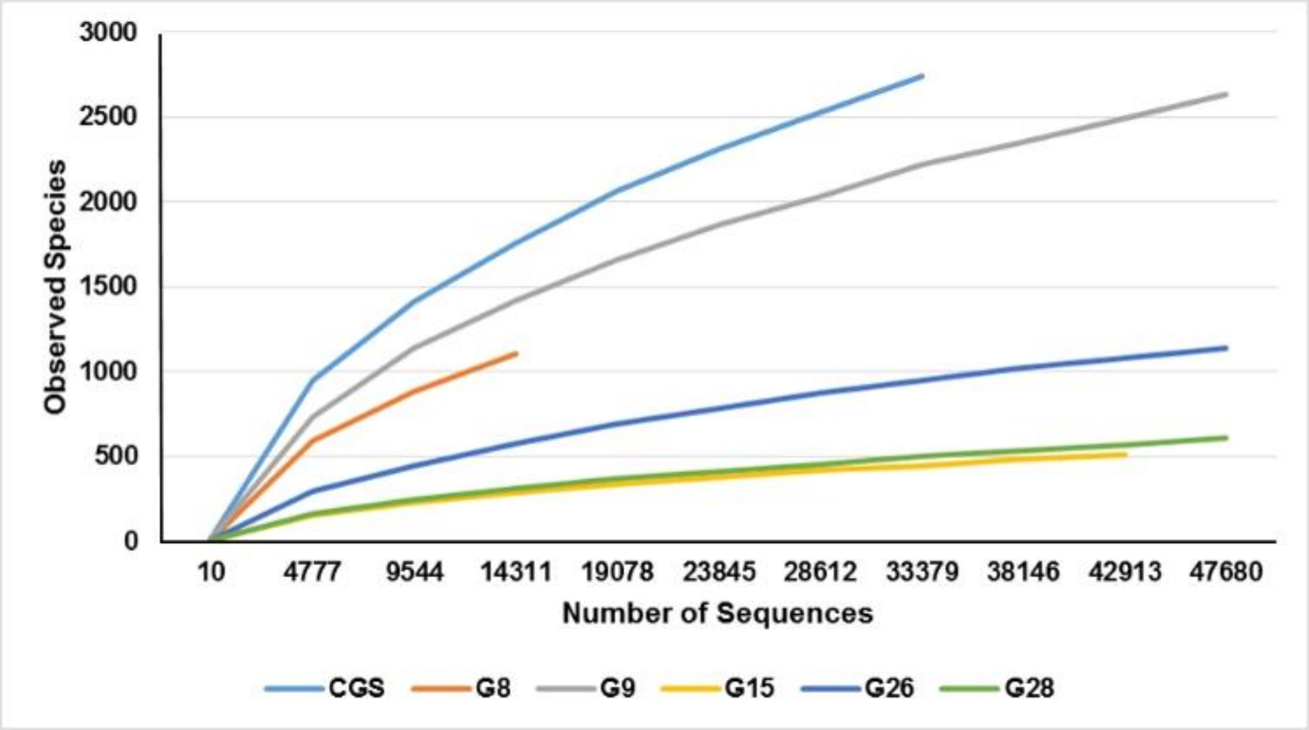


**Supplementary Figure-S6.** The rarefaction curve between observed OTUs (Operational Taxonomic Units) and number of Ion Torrent sequence.

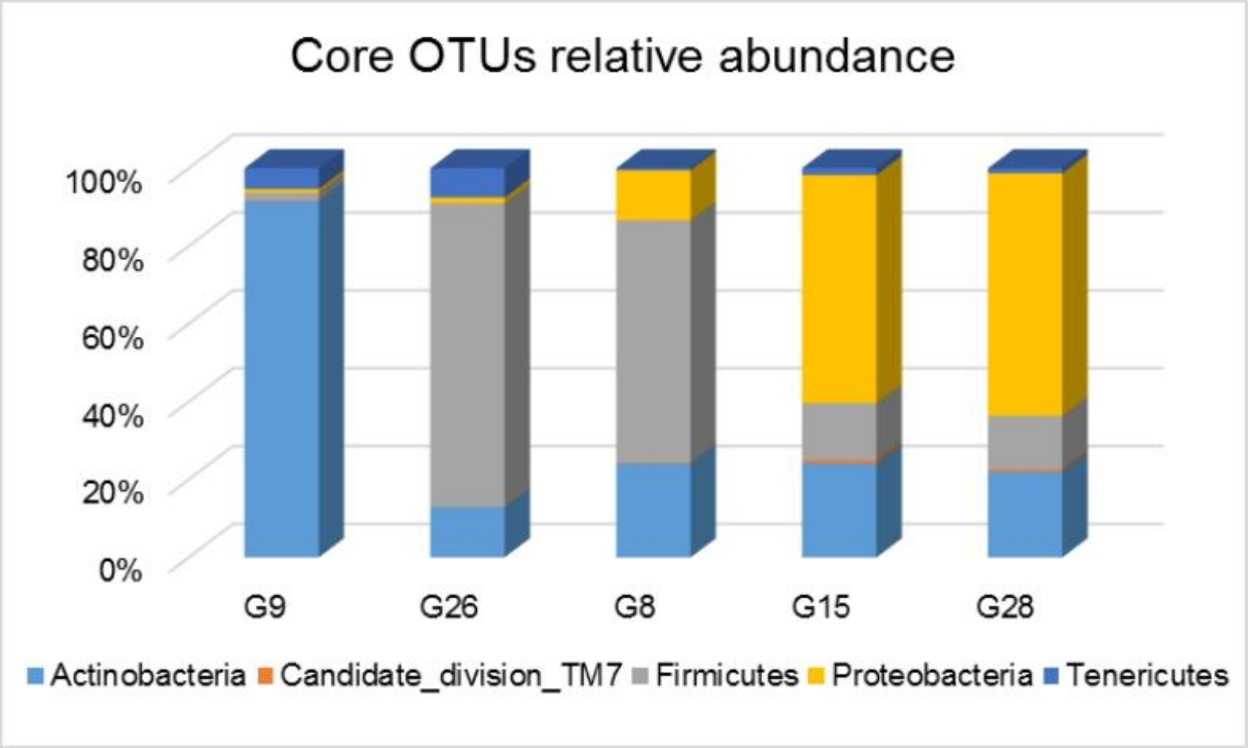


**Supplementary Figure-S7.** Relative proportions of different bacterial phyla constituting the ‘core microbiome’.

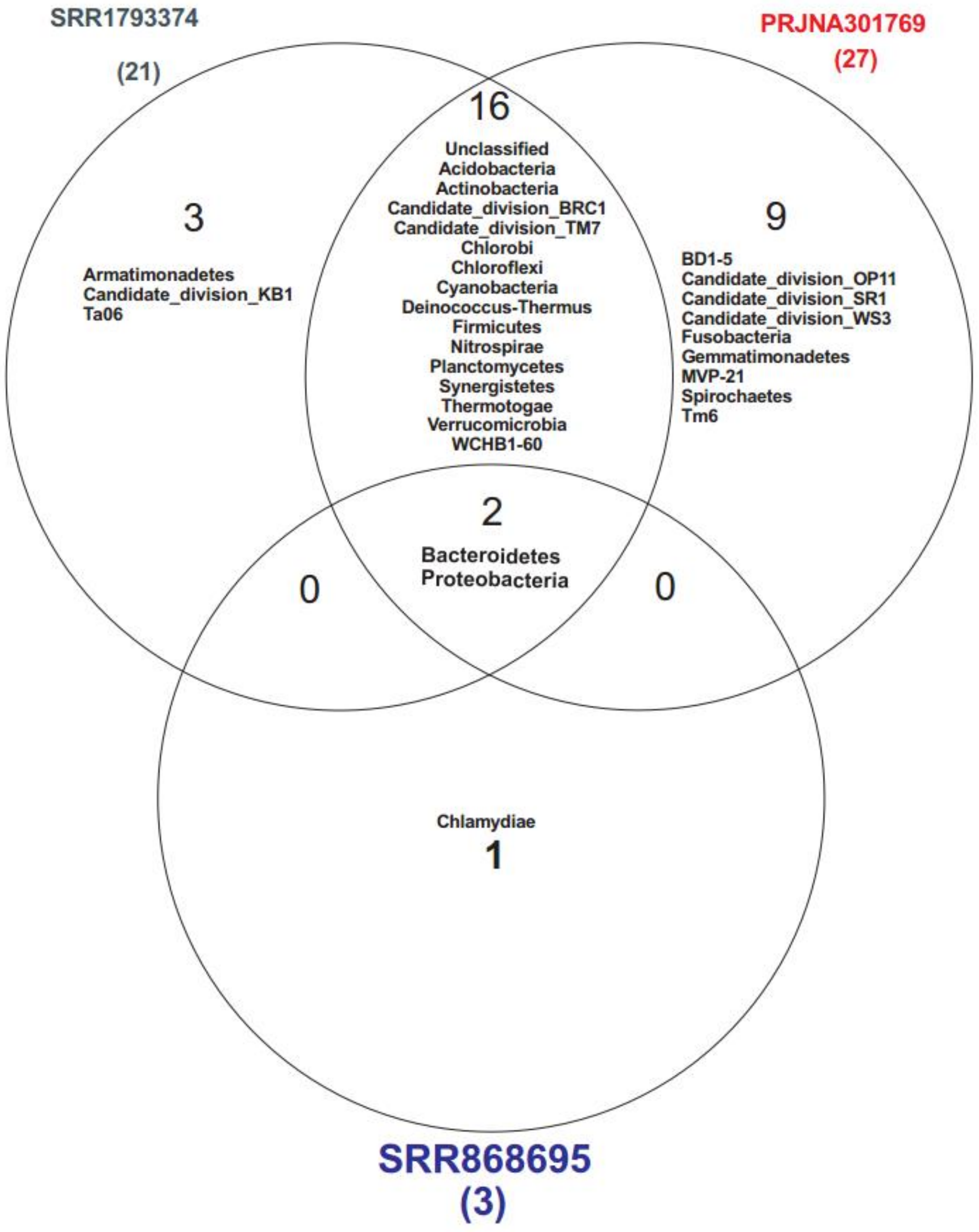


**Supplementary Figure-8.** The common bacterial phyla obtained in the comparison of three studies. SRR1793374 = DeMandal et.al.,2015; PRJNA301769 = This Study; SRR868695 = Veikkolainen et.al.,2014.
